# Pangenome graph analysis reveals evolution of resistance breaking in spinach downy mildew

**DOI:** 10.1371/journal.pbio.3003596

**Published:** 2026-01-20

**Authors:** Petros Skiadas, Melanie N. Mendel, Joyce Elberse, Guido Van den Ackerveken, Ronnie de Jonge, Michael F. Seidl

**Affiliations:** 1 Theoretical Biology and Bioinformatics, Department of Biology, Utrecht University, Utrecht, The Netherlands; 2 Translational Plant Biology, Department of Biology, Utrecht University, Utrecht, The Netherlands; 3 Plant-Microbe Interactions, Department of Biology, Utrecht University, Utrecht, The Netherlands; 4 AI Technology for Life, Department of Information and Computing Sciences, Utrecht University, Utrecht, The Netherlands; Duke University Medical Center, UNITED STATES OF AMERICA

## Abstract

Filamentous plant pathogens secrete effectors to successfully establish host infections. In resistant crop varieties, plant immunity can be triggered by immune receptors that recognize these effectors. Resistant crop varieties are grown in large-scale monocultures imposing strong selection pressure on pathogens, driving rapid evolution of effector repertoires resulting in the frequent breakdowns of resistance within just a few growing seasons. The oomycete *Peronospora effusa*, responsible for downy mildew on spinach, is an example of a rapidly adapting pathogen, but it is yet unknown how *P. effusa* can successfully overcome resistance of spinach by genomic adaptations. To close this knowledge gap, we here generated genome assemblies and constructed a pangenome graph for 19 isolates corresponding to 19 officially denominated resistance-breaking *P. effusa* races, which can cause disease on a differential set of spinach cultivars. Haplotype-resolved pangenome graph analyses revealed that many isolates emerged from recent sexual recombination, yet others evolved via prolonged asexual reproduction and loss of heterozygosity. By phasing effector candidates to determine their allelic variation, we identified effector candidates associated to resistance breaking of spinach varieties and reconstructed the evolutionary events that led to their diversification. The here developed and applied computational genomics approaches offer invaluable insights into the molecular mechanisms of the rapid evolution of *P. effusa*, and points to potential targets for future resistance breeding.

## Abstract 2

Plant pathogens secrete effectors to help establish host infections, but resistant crop varieties select strongly for resistance-breaking pathogens. This study of the oomycete pathogen responsible for downy mildew on spinach uses pangenome graph analyses of 19 resistance-breaking races to identify several potential effectors.

## Introduction

To establish a successful infection, filamentous plant pathogens secrete so called effector molecules that promote host colonization by, for example, circumventing or suppressing host immune responses [1,2]. Plant immunity can be activated in a gene-for-gene manner, where a single plant resistance protein is activated in response to a single pathogen effector, known by the term “avirulence factors,” leading to a strong hypersensitive response that stops pathogen infection, a process termed effector-triggered immunity [3,4]. In turn, pathogens that do not express, have mutations in, or have lost this particular effector can break the resistance of the host plant and thereby reestablish their virulence [5,6].

Effector-triggered immunity is qualitative, often monogenic or oligogenic, and thus it is a desirable trait for the development of resistant crop cultivars. In agriculture, these resistant cultivars are often deployed in large monocultures [7]. However, this agricultural practice exerts strong selective pressure on pathogens to overcome resistances, leading to the rapid diversification of pathogen populations [8,9]. On the genomic level, this strong selection drives the emergence of novel or the diversification of existing effectors to avoid host recognition and to re-establish successful host colonization [1]. Unsurprisingly, host resistances are often broken within only a few growing seasons after the introduction of resistant cultivars in the fields [10].

Oomycetes are a diverse and widespread group of filamentous organisms including many important pathogens of plants, causing devastating diseases and resulting in severe damage in agriculture and natural ecosystems [11,12]. To establish a successful infection, oomycetes utilize two classes of effector proteins: apoplastic effectors, which act outside the plant cells, and cytoplasmic effectors, which are translocated inside the plant cell [13]. Two main families of cytoplasmic effectors have been characterized in oomycetes thus far, the RXLR and Crinkler (or CRN) effectors [14,15]. These two effector families are characterized by the presence of conserved motifs at the N-terminus downstream of the signal peptide, which have been hypothesized to contribute to effector translocation into the host cell or to their secretion [16–19]. The C-terminal regions of these effectors vary significantly and are responsible for the effectors’ functions in the plant cell [13,18,20]. This region can be recognized by hosts’ immune systems, triggering hypersensitive response and host resistances [21]. Several nucleotide-binding leucine-rich repeat receptors (NLRs) have been identified that can recognize avirulence proteins, including the R3a *NLR* from a wild potato species that recognizes the AVR3a effector of the oomycete tomato and potato late blight pathogen *Phytophthora infestans* [21,22]. However, aside from effectors characterized in *Phytophthora*, *Hyaloperonospora*, and *Plasmopara*, studies assessing effector diversity across multiple isolates of the same species are limited in oomycetes, particularly in species outside these genera. The obligate biotrophic oomycete *Peronospora effusa* causes downy mildew on spinach, the economically most important disease of cultivated spinach worldwide [23,24]. This pathogen has been traditionally managed by fungicides and the extensive deployment of genetic disease resistances [25,26]. Resistant spinach cultivars, for example, those with the NLR *RPF1*, are thought to encode receptors that can recognize specific *P. effusa* effectors and thereby induce effector-triggered immunity [27]. These cultivars have been extensively used for spinach production and have been the most effective management tool for downy mildew, especially in organic practices [25]. However, *P. effusa* rapidly overcomes resistances in newly released spinach varieties, and currently no commercially available resistance remains effective. [24]. Due to this rapid evolution, a new *P. effusa* race is denominated every year based on isolates having the capacity to break spinach resistances, with 20 races denominated thus far [23,27,28]. Like many oomycetes, *P. effusa* is diploid and can reproduce both asexually and sexually [29]. Sexual reproduction between genetically divergent strains introduces new allelic combinations and increases heterozygosity, whereas prolonged clonal propagation can result in loss of heterozygosity through mechanisms such as mitotic recombination or gene conversion [23,30]. Consequently, isolates exhibit substantial variation in heterozygosity, with up to 4-fold differences [29]. Sexual recombination was suggested to be a powerful driver of the emergence of new *P. effusa* races [29,31]. However, we currently lack direct genomic evidence of recombination between the isolates or for the role of heterozygosity in the variation of the effector repertoires between *P. effusa* isolates.

*P. effusa* is one of the few oomycete species with multiple isolates having chromosome-level reference genome assemblies publicly available [32–34]. Additionally, we have recently developed a pangenome approach for in-depth comparisons of multiple genome assemblies and their structural annotations in depth [32]. Our previous comparison of six denominated races revealed that the genome of *P. effusa* is 57.8–60.5 Mb in size, is highly repetitive (56% repeat content), and is organized in 17 core chromosomes, with few isolates having a single additional accessory chromosome which is seemingly nonfunctional [32]. On average, of the 10,312 annotated genes, 472 are effector candidates that are often under positive selection and are highly variable between isolates [32]. While sexual recombination is suggested to play a role in the evolution of *P. effusa* [29,31], the level of recombination between the isolates has not yet been described. Additionally, while it is conceivable that effector variation between the *P. effusa* races is linked to the breakage of spinach resistances, no effector candidates associated with (a)virulence have been identified thus far. Here, we capitalized on our previously developed pangenome framework [32] to analyze chromosome-level genome assemblies for 19 denominated resistance-breaking *P. effusa* isolates. We further expand on that approach by phasing the genomes and analyzing the haplotypes in each isolate, revealing that while many *P. effusa* isolates are the result of recent recombination yet few also display patterns of prolonged asexual reproduction and loss of heterozygosity. Importantly, phasing of all effector candidates enabled us to identify few effectors that can be associated to the breaking of spinach resistance and enabled us to reconstruct the molecular processes that shaped their genomic variation. This provides invaluable insights into the mechanisms behind the rapid evolution of *P. effusa* and for the first time identifies targets for future experimental analysis to uncover their functions and roles in resistance breaking as well as their application as tools to accelerate resistance breeding.

## Results

### *Peronospora effusa* genomic variation is due to the expansion of repeats and gene copy numbers

**Fig 1:**
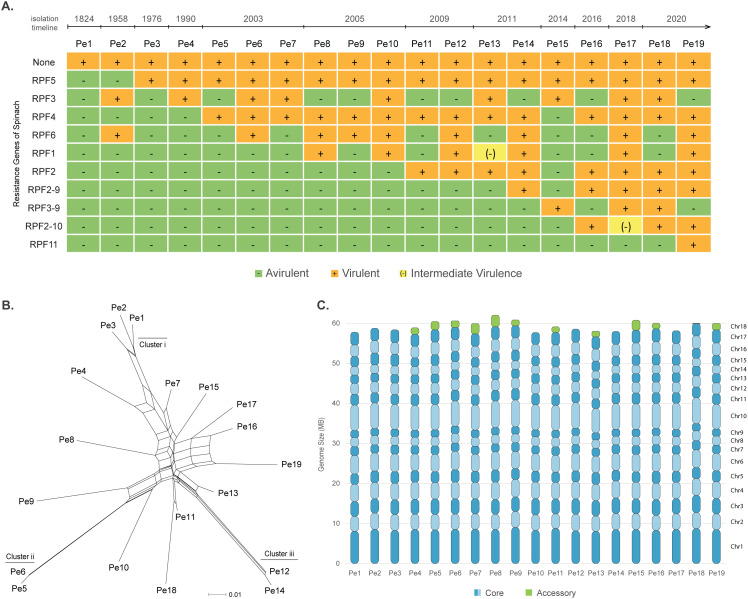
*Peronospora effusa* isolates break various spinach resistances. **A.** The (a)virulence phenotypes of 19 *P. effusa* isolates that break different combination of spinach resistance genes. The year of identification is indicated as well as the NLR resistance genes present in the spinach cultivars that are successfully infected by the virulent *P. effusa* race. **B.** Neighbor-net phylogenetic network indicates the relationships between *P. effusa* isolates. Branch lengths are proportional to the calculated number of substitutions per site. The parallel edges connecting different isolates indicate conflicting phylogenetic signals, suggesting recombination between isolates (Data in Zenodo). **C.** Stacked bar plots display the different chromosomes and their respective sizes for each *P. effusa* isolate. The presence and size of the accessory chromosome 18 is indicated in green (Data in S2 Table).

To explore the genomic variation in *P. effusa*, we selected 19 isolates representing 19 races denominated for their capacity to break different combination of resistance alleles introduced in spinach (Fig 1A) [35]. Like other oomycetes, *P. effusa* has both a sexual and an asexual reproduction cycle [29]. To better represent this complex phylogeny, we determined the relationship between the 19 isolates with a neighbor-net phylogenetic network analysis using 200,934 biallelic single-nucleotide polymorphisms (SNPs). Next to few clusters of closely related isolates, we observed that isolates that are branching from the center of the phylogenetic network are likely the result of a recent recombination between distant isolates (e.g., *Pe7*, *Pe11*, or *Pe13;* PHI-test <0.0001 statistically supports recombination between isolates). Moreover, most isolates have long branches due to a high number of unique SNPs (on average 6,605 SNPs or 3.9% of the SNPs are unique per isolate), suggesting prolonged asexual reproduction or recombination with closely related *P. effusa* genotypes that have not been isolated and analyzed (Fig 1B).

Of the 19 *P. effusa* isolates, genomes of six have been previously assembled, which revealed 17 highly conserved and collinear core chromosomes and an additional 18th accessory chromosome that is present in few isolates [32]. Here, we generated genome assemblies for the remaining 13 isolates using Nanopore and Illumina sequencing data and created chromosome-level, haploid genome assemblies with total genome assembly sizes ranging between 57.8 and 62.1 Mb (Fig 1C; S1 and S2 Tables). Twelve out of the 19 genome assemblies have few contigs that share similarity with the previously described accessory chromosome 18 [32], which varies greatly in size and fragmentation (0.08–2 Mb; one to six contigs). The fragmentation is a direct result of the highly repetitive nature of chromosome 18, and the size difference corroborates our previous findings suggesting chromosome 18 degrading (S2 Table) [32]. Seven isolates have only contigs matching the 17 core chromosomes. All contigs that match the 17 core chromosomes have telomeric repeats with the repeat motif ‘TTTAGGG’ on both ends, suggesting that we successfully obtained complete and chromosome-level genome assemblies for all 19 isolates. The assembly completeness, evaluated by Benchmarking Universal Single-Copy Ortholog (BUSCO) analysis using the Stramenopiles database (v. odb10), revealed a 99% BUSCO completeness score, which is comparable with previous chromosome-level *P. effusa* genome assemblies [32,36], indicating that these assemblies are highly contiguous and successfully captured most protein-coding regions.

To evaluate the collinearity of the 17 core chromosomes, we performed whole-genome alignments of the 19 genomes based on sequence similarity and on relative position of protein-coding genes. The genome assemblies are nearly completely co-linear, except for few intrachromosomal rearrangements on chromosomes 1, 3, 10, and 17 (S1 Fig). These findings corroborate previous observations that the core chromosomes of *P. effusa* are conserved with no gross chromosomal rearrangements [32,34].

**Fig 2:**
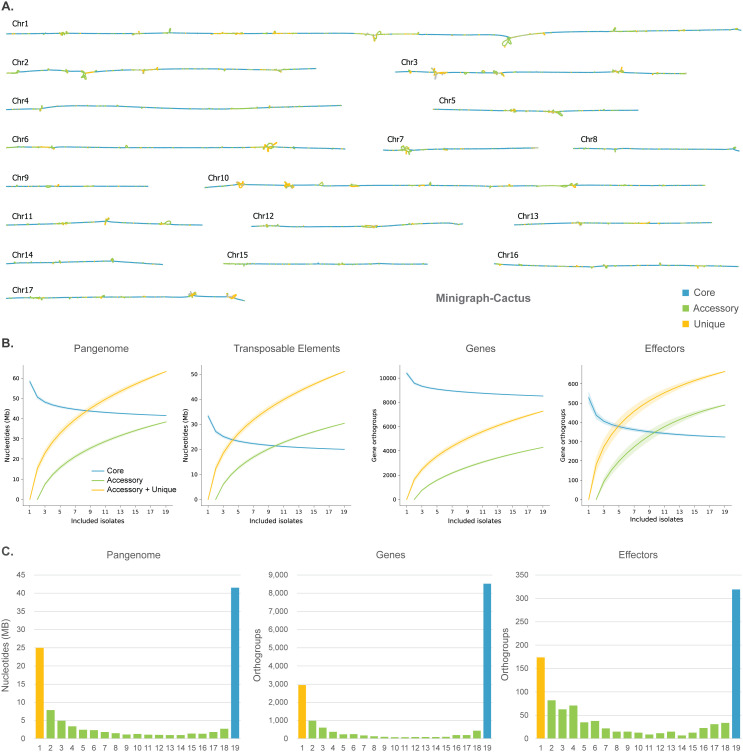
Pangenome graph analysis for 19 *Peronospora effusa* isolates. **A.** Pangenome graphs of the 17 core chromosomes that are present in all *P. effusa* isolates were created with Minigraph-Cactus and visualized with Bandage [37,40] (Data in Zenodo). **B.** Saturation plots based on the pangenome graph on the nucleotide level for the whole genome and transposable elements, as well as on the gene orthogroup level for all genes and effector candidates (Data in Zenodo). **C.** Bar plots show the total size of pangenome graph nodes, the total number of genes, and the total number of effectors that belong to one to 19 isolates (Data in S7 Table).

To systematically describe the genomic variation of these 19 *P. effusa* isolates, we utilized our previously developed pangenomic approach to directly compare the chromosome-level assemblies without possible reference biases [32]. We used all 19 assemblies to create 17 sequence-resolved pangenome graphs, one for each core chromosome, based on Minigraph-Cactus [37]. The combined pangenome graph has 3,570,190 nodes and 4,858,391 edges, which is only a small fraction of all theoretically possible connections between the nodes, indicating that the addition of genomes does not lead to a complex pangenome structure. Most nodes have only two connections, indicating a mostly linear graph, while only 82 nodes have a degree of 10 or higher (S2A Fig). The total size of the combined graph is 104.8 Mb, 80% larger than the average *P. effusa* genome assembly (58 Mb), suggesting that while most of the genome organization is conserved, the pangenome graph nevertheless captures a significant amount of accessory genomic regions (Fig 2A).

Consistent structural annotation is essential to discover and describe genomic variation between isolates. Following our previous methods, we used a transposable element (TE) library, based on multiple *P. effusa* isolates, to annotate TEs, resulting in 54–57% of each genome being annotated as TEs (S3 Table) [32]. For protein-coding gene annotation, we took advantage of our previously developed joined structural annotation approach [32], which resulted in the annotation of 9,571–10,540 protein-coding genes for each isolate. The joined structural annotation added between 239–866 genes (2.4%–9.0%) that have been missed by the conventional approach that annotated each isolate independently. We assigned these genes to orthologous groups based on their relative position on the pangenome graph, creating 15,740 syntenic orthogroups, with each isolate being either absent or represented by a single gene in each group. To identify effector candidates, we then searched the predicted protein-coding genes and additional open reading frames for those encoding proteins with a predicted signal peptide and for RXLR and CRN amino acid motifs, which yielded 354–475 putative RXLR and 33–66 putative CRN effectors per isolate (S3 Table), abundances similar to those in previously assembled *P. effusa* isolates [32,34].

The overall genomic variation between the 19 *P. effusa* isolates can be uncovered by directly querying the pangenome graph. This analysis revealed that 39.6% (around 41.5 Mb) of the *P. effusa* pangenome is conserved, 36.6% (38.3 Mb) is found in two or more isolates, and 23.8% (24.9 Mb) is unique for single isolates (Figs 2A and S2C). Thus, for each isolate on average 71.2% of the genome is core, 26.5% is accessory, and 2.2% is unique (S2C Fig). When we compared this to the previously generated pangenome based on only six isolates [32], we observed that the percentage of core regions had a small decrease (by 9.5%), the accessory regions greatly increased (49.4%), while the unique regions decreased (37.2%). This was expected, since the core regions of six isolates remain largely conserved in the 19 isolates, while the additional isolates add context to the shared variation between *P. effus*a isolates, overall resulting in the unique regions to decrease in relative abundance and the accessory to increase. Unexpectedly though, the unique regions per isolate still represent a significant portion of their genomes (1.6%–4.1%), causing the total size of unique regions in the 19 isolate pangenome to double in comparison to the six-isolate pangenome (1.25–2.50 Mb), suggesting that there is significant genomic variation yet to be discovered (S2C Fig).

The abundance of genomic variation is also visible in the saturation plots, which reveal an open pangenome in *P. effusa*, especially for TEs and effector genes (Fig 2B). The pangenome node distribution has a distinct U-shape characteristic of most pangenomes, with most regions either being present in all isolates (core) or unique to one isolate [38,39]. The distribution is also skewed towards lower frequency modes, suggesting that most genomic variation in *P. effusa* mainly arises from recent sequence expansions present in few isolates (Fig 2C). In *P. effusa*, genome expansions have been previously associated with the activity of TEs [32], but genes and especially effectors exhibit similar pangenome node distribution (Fig 2C), suggesting that gene and effector variation is most likely caused by gene copy-number expansions and novel mutations rather than by deletions.

### Phasing of genomic variation correlates with heterozygosity

**Fig 3:**
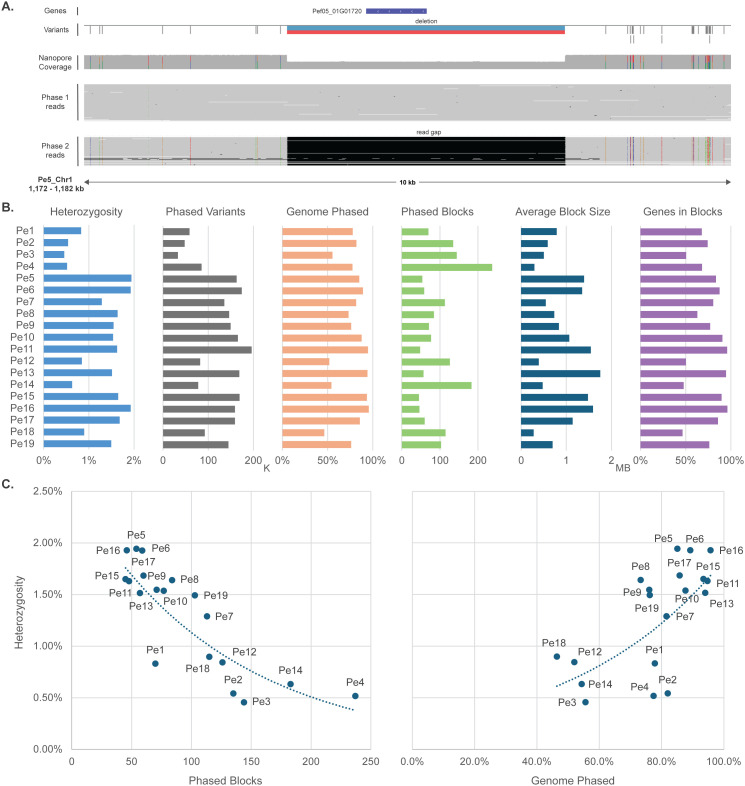
Phasing of *Peronospora effusa* genomes based on variant calling with Nanopore reads. **A.** Example of a heterozygous region in chromosome 1 of *Pe5* visualized with IGV [44]. From top to bottom, the panel shows an annotated gene in the heterozygous region, the variants called from PEPPER with gray lines and the deletion from Sniffles2 with red and blue, the coverage of Nanopore reads, the mapped Nanopore reads that are grouped as phase1, and the Nanopore reads of phase2 where most heterozygous SNPs and the reads gap appear (Data in Zenodo and NCBI). **B.** Bar plots show the differences in phasing results for the 19 *P. effusa* isolates. It reports the genome-wide heterozygosity (variable nucleotides divided by total genome size), the number of phased variants (scale in thousand variants), the percentage of the genome that is phased (total phase block size divided by total genome size), the number of phased blocks, the average size of phased blocks (scale in million bases), and the percentage of genes that fully overlap with phased blocks (Data in S7 Table). **C.** Scatterplots show the association between the level of heterozygosity of the 19 isolates and the number of phased blocks (left) or the percentage of the genome that is phased (right) (Data in S7 Table).

Like many other oomycete species, *P. effusa* is a heterozygous, diploid organism [41]. Thus, phasing of the two haplotypes in the genomes is needed to fully account for the observed genomic variation. Due to the obligate biotrophic nature, we do not have access to the parental genomes, and thus it is challenging if not impossible to separate phases (haplotypes) prior to genome assembly. We therefore used a combination of computational approaches based on Nanopore long reads to perform variant calling for each of the 19 genome assemblies and to subsequently phase heterozygous variants. First, variant calling was performed using PEPPER, which phased the Nanopore reads and discovered phased short variants (1–59 bp) [42]. Then, the phased long reads were used with Sniffles2 to discover phased larger insertions and deletions (4–40,000 bp) [43] (Fig 3A). For each isolate, we discovered on average 126,742 heterozygous phased variants, of which the vast majority (98.5%) were identified by PEPPER. Due to their larger size, however, Sniffles2 contributes 72%–86% of the variable nucleotides in each genome assembly. The allele frequency of these variants follows a normal distribution with a median close to 0.5 for all isolates, as expected for a diploid organism (S3 Fig). Importantly, the large number of phased variants and the length of the Nanopore reads (on average a N50 of 28 kb) allowed for the creation of large, phased blocks (1 Mb on average), up to the full size of complete chromosomes (Fig 3B).

Heterozygosity, and thus the number of phased variants, varies greatly between the isolates (0.46%–1.94% heterozygosity and 32,667–196,278 variants) (Fig 3B). These differences are notable at every aspect of the genome phasing procedure, with highly heterozygous isolates (>1.5%) having less and longer phased blocks (45–77 blocks) that cover most of the genome (>85%), while isolates with lower levels of heterozygosity (<0.9%) have more and smaller sized blocks (115–237 blocks) that cover only about half of the genome (46%–55%) (Fig 3B). Consequently, there is a clear negative correlation between the level of heterozygosity and the number of phased blocks and a positive correlation between heterozygosity and the percentage of genome phased (Fig 3C). These observations similarly apply to individual chromosomes, whose heterozygosity varies greatly, both between chromosomes in one isolate as well as between corresponding chromosomes in different isolates. For example, chromosome 13 is almost entirely homozygous in *Pe12* (0.08% heterozygosity), while in *Pe11* it is heterozygous (1.48% heterozygosity). Inversely, chromosome 11 is heterozygous in *Pe12* (2.29% heterozygosity) and mostly homozygous in *Pe11* (0.77% heterozygosity). Thus, the number and coverage of phased blocks vary greatly between chromosomes, and it was not possible to fully phase all chromosomes in even the highly heterozygous genomes. Nevertheless, our approach still enabled us to analyze the heterozygosity of all isolates in depth, and we were able to successfully phase smaller chromosomes that are highly heterozygous.

**Fig 4:**
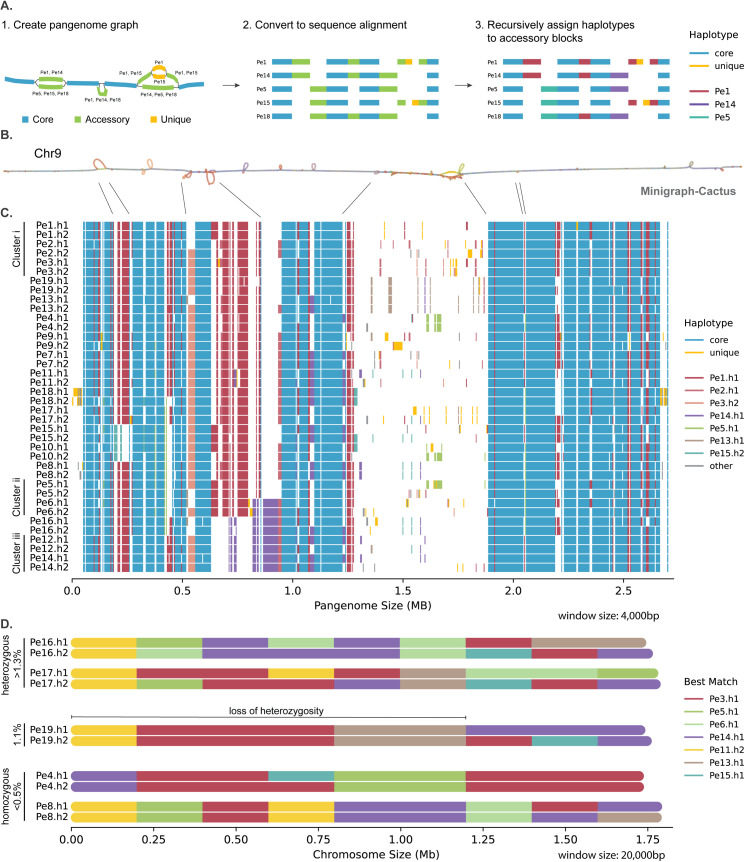
Haplotype comparison of the fully phased chromosome 9 uncovers a variety of evolutionary processes. **A.** Explanation of the here applied approach to assign haplotypes to accessory nodes of the pangenome and subsequent visualization: 1. we create a pangenome graph and parse its variation; 2. the graph is transformed into a sequence alignment for visualization; 3. the core and unique nodes remain unchanged, while the accessory nodes are recursively assigned to different haplotypes. The order and the color of the assigned haplotypes is shown in the figure legend. **B.** Pangenome graph of the phased chromosome 9 created with Minigraph-Cactus and visualized with Bandage [37,40]. The nodes of the graph are coloured based on the haplotypes assigned in C (Data in Zenodo). **C.** Alignment of haplotypes of chromosome 9 for all phased *P. effusa* isolates, derived from the pangenome graph in 4 kb windows, as described in A (Data in Zenodo). **D.** For both phases of five isolates, we coloured chromosome 9 based on the best match to a different isolate in a 20 kb window. As targets, we selected the seven isolates that contributed the most to genomic diversity (Data in Zenodo).

*Peronospora effusa* haplotypes reveal sexual and asexual evolutionary pathways. To compare any genomic region between the 19 isolates, phased or unphased, from a single gene to a full chromosome, we utilized the variation that is directly captured in the pangenome graph. We divided the nodes of the pangenome graph into windows (size specified in each figure) and assign different haplotypes based on the observed variation. In order to not overemphasize small amounts of variation, if all isolates are more than 95% identical in a window, the window was assigned to the core, but if the variation was higher than 5% and the window was only found in one isolate, the window was assigned as a unique genomic region. The windows with accessory variation (>5%) were recursively assigned to isolates based on their genomic relationship (Fig 1B) (the haplotype order is shown in the corresponding figure legend). For *P. effusa*, we have previously shown that most genomic variation in the sequenced isolates can be observed when comparing isolates that are belonging to the three assigned clusters (cluster i–iii) [29]. We thus assigned windows of variation first to the isolates from clusters i–iii, and then iteratively to the isolates that are located closer to the center of the phylogenetic network (e.g., *Pe11* or *Pe13*). We then coloured the nodes in each window based on their assigned haplotype, thus creating haplotype blocks that could be visualized based on their respective position in the pangenome graph (Fig 4A).

This method was applied to a fully phased chromosome to capture all genomic variation in each isolate, and through their comparison, to discover possible signatures of recombination and differences in the evolution of *P. effusa* isolates. As an example, we focused on chromosome 9 as it is the smallest *P. effusa* chromosome, is highly conserved, and has a low TE content [32]. Additionally, it has been fully phased for most isolates (85%–100% for 11 isolates) and the remaining isolates are either near homozygous (<0.3% heterozygosity for *Pe3*, *Pe4*, *Pe7*, *Pe8*, and *Pe14*) or are overall highly heterozygous but have lost heterozygosity in large regions of the chromosome (*Pe12*, *Pe18*, and *Pe19*). With the phased chromosome 9 from these 19 isolates, we created a pangenome graph with Minigraph-Cactus [37] (Fig 4B). The pangenome graph of the phased chromosome 9 has 104,800 nodes and 143,918 edges, is highly linear, and 2.7 Mb long: 52% longer than the average size of chromosome 9 and 16.4% longer than the pangenome graph of the unphased chromosome 9 (67,084 nodes and 91,869 edges). Thus, phasing of this chromosome captured additional genomic variation that was not present in the pangenome graph that is solely based on the haploid genome assemblies.

By comparing the different combinations of haplotype blocks on chromosome 9, two main genomic organizations emerged, namely the one observed in cluster i (*Pe1*, *Pe2,* and *Pe3*) and the other one in cluster iii (*Pe12* and *Pe14*). Most haplotype blocks from cluster i that are shared with isolates outside the cluster, are present in *Pe2* and *Pe3*, which suggests that in most cases other isolates have recombined with *Pe2* and *Pe3* rather than the much older *Pe1* isolate. Additional haplotype blocks can be found in a small number of isolates, and these were assigned to *Pe5*, *Pe13*, and *Pe15*. The observed recombination of these haplotype blocks can explain the chromosomal markup of any other isolate, with the addition of few unique blocks, providing strong support of sexual recombination (Fig 4C).

Potential recombination between isolates can be more clearly described by dividing each chromosome into multiple windows and comparing these directly between the isolates, thereby revealing the variation between the phases of different isolates. To this end, we split each chromosome in 20 kb windows and exploited the pangenome graph to assign to each window the best match from a different isolate (Fig 4D). To ease interpretation, we selected one representative isolate from each phylogenetic branch from a hierarchical clustering based on the accessory nodes of the pangenome graph. Note, however, that these are not always identical to the isolates selected when assigning haplotypes (S4 Fig). The combination of different matches in a chromosome offers evidence of past recombination events, while the difference between the phases can identify the timeline of those events.

Based on this approach, we selected five distinct examples of isolates to showcase the different processes that contributed to their evolution: *Pe4* is a clear example that demonstrates the occurrence of recombination. It clusters close to isolates from cluster i, but its mitochondrial phylogeny is the same with the isolates in cluster ii. Moreover, *Pe4* also has the accessory chromosome 18, which is not present in isolates from cluster i [29,32]. Evidence of recombination is found in the variation of chromosome 9 of *Pe4* that is most similar to *Pe3*, with some regions matching to *Pe5* and *Pe14* (Fig 4D). Like *Pe4*, *Pe8* is positioned in the middle of the phylogenetic network, has the accessory chromosome 18, and shows clear evidence of recombination as chromosome 9 matches to clusters i, ii, and iii (Figs 1B and 4D). While both *Pe4* and *Pe8* are the result of recombination, they show low levels of heterozygosity throughout the chromosome (0.49% and 0.29%, respectively), suggesting an extended period of asexual reproduction, which is further corroborated by a high number of unique SNPs (*Pe4* and *Pe8* have 7,031 and 9,841 unique SNPs, respectively) (Fig 1B). In contrast, isolates like *Pe16* and *Pe17* have much higher levels of heterozygosity (1.49% and 1.29%, respectively), and display clear evidence of recent recombination events, since their best matches to other isolates differ greatly between their two alleles (Fig 4D). The recent recombination is also evident in the lower number of unique SNPs of *Pe16* (5,740 unique SNPs). However, *Pe17* has 7,730 unique SNPs, suggesting that there might be additional isolates closely related to *Pe17* that are not part of our analyses (Fig 1B). *Pe19* also shows evidence of recombination from distant (*Pe3* and *Pe14*) and closely related isolates (*Pe11*, *Pe13*, and *Pe15*) (Fig 4D) and has 12,528 unique SNPs (Fig 1B). While *Pe19* is heterozygous (1.07%), this variation is in the last third of the chromosome, while the first two-thirds show a complete loss of heterozygosity (Fig 4D).

These haploblock recombinations have been showcased here on a single chromosome, but our observations can be generalized for the nearly complete genomes of our collection of isolates. While we could not fully phase the entire genome, the approach of assigning haplotypes based on the best matches between chromosomes can also be applied to the haploid, unphased genomes, which also provided clear evidence of recombination. We observed two groups of isolates: first, isolates that belong to clusters i–iii that share most haplotypes with isolates from within their respective cluster. The second group of isolates display a mosaic pattern of best matches to different isolates (S5 Fig). This pattern, with chromosomes being formed by complex combinations of genomic regions from many different lineages, suggests that isolates outside of the three clusters are the result of multiple recent sexual recombination events, which is further corroborated the PHI-test for recombination (*p* < 0.0001). Our observations from both unphased haploid and phased chromosomes therefore highlight that *P. effusa* isolates can evolve by a range of evolutionary processes. It is conceivable that these processes underlie the rapid emergence of new aggressive *P. effusa* races that can break host resistances, however, further experimental evidence is needed to fully support this statement.

### Changes in virulence are the result of multiple independent evolutionary adaptations

Variation in effector genes between the 19 *P. effusa* isolates is most likely responsible for resistance breaking in spinach (Fig 1A). Our phased genome assemblies enable us for the first time to explore the full extent of gene and protein variation between *P. effusa* races. We searched for phased variants overlapping with protein-coding genes and integrated them into the gene sequence, thus, when possible, creating two alleles for each gene. For each isolate 2,916–35,426 phased variants overlapped with genic regions, affecting between 1,066 up to 5,952 genes, including seven to 101 gene deletions (S4 Table). Despite the large number of heterozygous variants that overlapped with genes, for each isolate 94% to 99% of all their proteins are more than 99% identical between the two alleles (S6 Fig). Interestingly, while most alleles are highly similar, genes encoding secreted proteins, RXLR and CRN effectors, and especially genes encoding proteins of the same functional group that are located next to each other in the genome (clustered genes) are enriched for non-synonymous allelic variation (S7 Fig).

While protein variation is limited between the haplotypes of each isolate, the variation across all haplotypes of all isolates is significant. The presence and absence of genes can be directly inferred from the number of accessory or unique orthogroups, which revealed that 88% of all proteins have an ortholog present in all isolates. Significant genomic variation can also be present in core orthogroups due to mutations, especially when these mutations lead to frame shifts or premature stops. To investigate this, we compared the protein sequence identity in each orthogroup, which revealed that on average 23% of all proteins in each isolate have identical orthologs in both alleles for all *P. effusa* isolates, while half of all proteins have all orthologs in all isolates at least 60% identical in sequence (S8 Fig). Importantly, half of all proteins in an isolate miss a similar ortholog (<60% sequence identity) in at least one allele of at least one isolate, considerably more than the 12% observed just from the gene presence/absence variation. Effector proteins display a similar trend compared with other protein-coding genes, but are more variable (11% identical orthologs, 32% orthologs >60%), while effector proteins that derived from genes that are not part of a physical cluster of effector genes are more conserved (15% identical orthologs, 39% orthologs >60%) (S8 Fig). These observations are expected since clustered effector genes are the result of gene copy-number expansions and evolve under positive selection [32].

We have previously shown that unclustered effectors do not typically evolve via gene copy-number expansions, and these genes lack highly similar paralogs in the genome [32]. Consequently, we argued that haplotype variation of single unclustered effectors could be directly connected to the phenotypic differences in virulence between the 19 isolates (Fig 1A). To test this hypothesis, we searched for effectors that can be associated with avirulence on each host resistance gene (Fig 1A), assuming that the effector protein would need to be highly conserved in all avirulent isolates, and inversely, the gene encoding the effector protein should be absent or significantly changed in all the virulent *P. effusa* isolates. For each spinach resistance gene, our approach yielded 20–50 effector candidates that were manually inspected, resulting in the identification of four effector candidates we will further discuss to highlight different evolutionary processes that could lead to the emergence of resistance-breaking *P. effusa* isolates.

**Fig 5:**
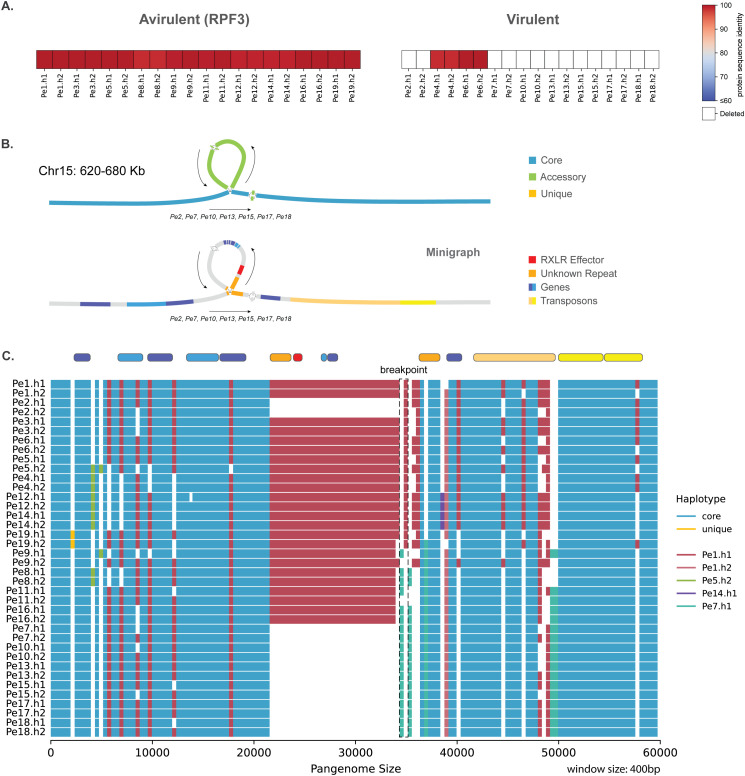
The deletion of a single RXLR effector gene correlates with the resistance breaking of *RPF3.* **A.** Protein sequence similarity to the longest effector of the Chr15-316 orthogroup, which includes both alleles of all isolates separated by the (a)virulence phenotype on the spinach with the resistance gene *RPF3* (Data in Zenodo). **B.** Pangenome graph of a 60 kb region around the RXLR effector shows the variation in this region and the annotated genes and TEs (Data in Zenodo). **C.** Full alignment of the haplotypes in this region based on the approach explained in Fig 4A, using a 400 bp window. The gene and TE annotations for *Pe1* are visualized on the top (Data in Zenodo).

Spinach cultivars with the *RPF3* gene are resistant to 10 of the 19 isolates. We identified a single 140 amino acid long effector with a RXLR-EER motif, whose gene is localized in the middle of the large chromosomal arm of chromosome 15, that is highly conserved in 12 isolates (≥98.3% protein sequence identity of both alleles) and absent in seven isolates. This pattern correlates with the *RPF3* resistance, with all seven isolates that lack this effector being virulent on *RPF3* spinach (Fig 5A). Pangenome-enabled comparison of the region around this gene revealed a deletion in a 12.7 kb region that includes three annotated genes and two identical repetitive regions at the start and end of this region, annotated as unknown TEs (Fig 5B). The isolates that lack this genomic region have only one copy of this TE, suggesting that the presence or activity of the TE contributed to the deletion of this region. The analyses of the haplotype blocks of this region reveals that six of the isolates with the deletion are closely related (*Pe7*, *Pe10*, *Pe13*, *Pe15*, *Pe17*, and *Pe18*) and possibly share a common ancestor. While *Pe2* also contains this deletion, it has a haplotype distinct from these six isolates and is more closely related to isolates without the deletion (*Pe1*, *Pe3*, and *Pe6*), while one of the deletion breakpoints is unique for *Pe2* (Fig 5C). These observations collectively indicate that the observed deletions of the region occurred in two separate events.

**Fig 6:**
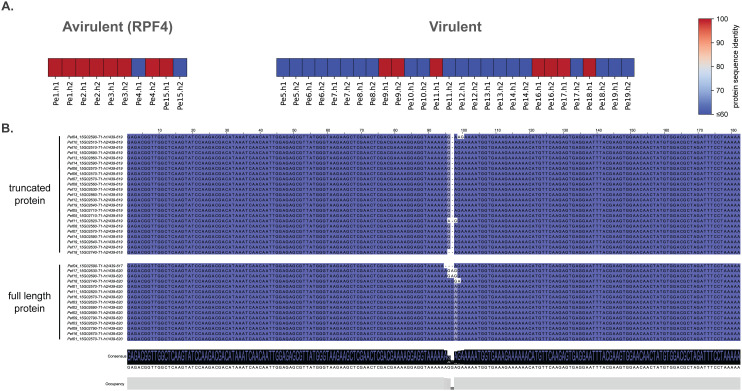
A single mutation in a RXLR effector gene associates with the resistance breaking of *RPF4.* **A.** Protein sequence similarity to the longest effector of the Chr15-16 orthogroup, which includes both alleles of all isolates, separated by the (a)virulent phenotype against the spinach with the resistance gene *RPF4* (Zenodo). **B.** Nucleotide alignment of a 182 bp long region of the effector genes belonging to Chr15-16 orthogroup separated by the protein haplotype (truncated vs. full length).

Spinach cultivars with the *RPF4* gene are resistant to five of the 19 isolates. We identified an effector gene encoding a protein with RXLR-EER and WY motifs, localized on chromosome 15, which is almost identical for 15 alleles (>99.6% protein sequence identity), while the other 23 alleles have only 30.0% identity, 21 of which can be found in isolates that are virulent on *RPF4* spinach (Fig 6A). This is the result of a single nucleotide deletion of the 535th nucleotide of the gene, which causes a frameshift, a downstream premature stop, and thus a truncated protein. As a result, the truncated protein is only 219 amino acids long, much shorter than the 720 amino acids long protein encoded by the allele in avirulent isolates, thus missing the entire C-terminal region of the effector that typically is functional in plant cells [13,18,20]. Notably, this is the only example where a single mutation can be attributed to all variation of this gene between the isolates.

**Fig 7:**
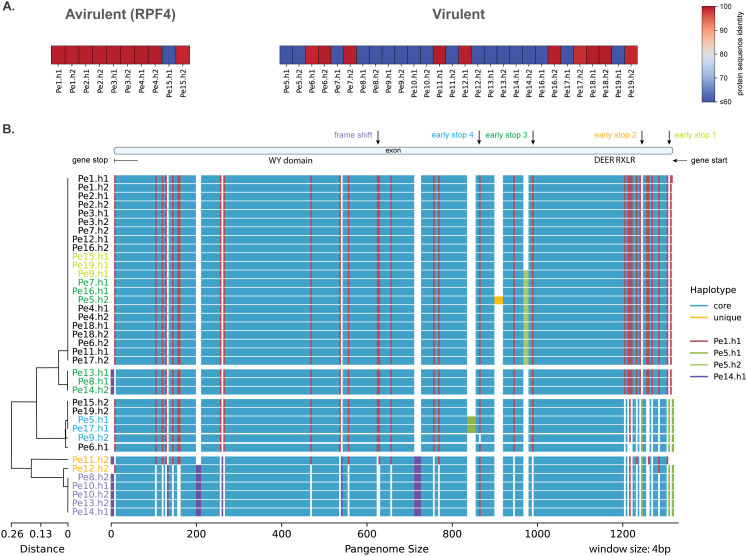
Mutated RXLR effector gene associates with the resistance breaking of *RPF4.* **A.** Protein sequence similarity to the longest effector of the Chr5-429 orthogroup, which includes both alleles of all isolates, separated by the (a)virulent phenotype against the spinach with the resistance gene *RPF4* (Data in Zenodo). **B.** Full alignment of the haplotypes in this region based on the method on Fig 4A, using a 4 bp window and ordered based on the phylogeny of this region. Isolates are coloured based on the gene mutations or in black for the virulent phenotypes. The gene structure for *Pe1* and the mutated positions that lead to the truncated proteins are visualized on the top (Data in Zenodo).

Virulence on *RPF4* spinach varieties can also be associated with a second effector, 387 amino acid long, with the RLXR-EER and WY motifs, whose encoding gene is localized on chromosome 5. Here, we observed a highly conserved haplotype (>98.2% protein sequence identity) in avirulent isolates and haplotypes in virulent isolates that are either only half the size or completely deleted (Fig 7A). These nonfunctional haplotypes resulted from five separate mutations in the gene, causing early stops in the open reading frame. The appearance of these mutations in the isolates is consistent with the phylogeny of this gene, indicating that there are up to five pseudogenisation events (Fig 7B). Beyond these five mutations, we observed an accumulation of mutations in the gene in the virulent genotype that possibly followed the pseudogenisation events.

**Fig 8:**
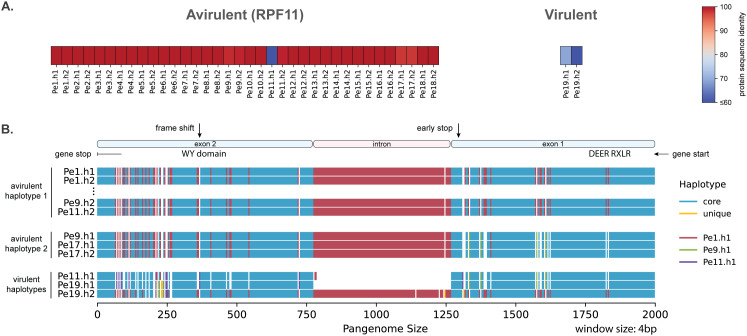
A mutated RXLR effector gene associates with the resistance breaking of *RPF11.* **A.** Protein sequence similarity to the longest effector of the Chr13-220 orthogroup, which includes both alleles of all isolates, separated by the (a)virulent phenotype against the spinach with the resistance gene *RPF11* (Data in Zenodo). **B.** Full alignment of the haplotypes in this region based on the method on Fig 4A, using a 4 bp window and grouped for virulence against the spinach gene *RPF11*. The gene structure for *Pe1* and the mutated positions are visualized on the top (Data in Zenodo).

*RPF11* resistance is broken only by *Pe19* and there is only a single 660 amino acid long effector that is conserved in all other isolates but *Pe19*. The two avirulent haplotypes share at least 97.7% sequence identity, while the virulent haplotypes are 36%–56% shorter in length, thus being likely nonfunctional (Fig 8A). These truncations are the result of two different mutations, one SNP creating a premature stop codon that is shared between *Pe19*.h1 and *Pe11*.h1 and one frameshift that causes an early stop codon in *Pe19.h2*. Interestingly, we discovered that the intron is no longer present in *Pe19*.h1 and *Pe11*.h1. These two haplotypes share many haplotype blocks, and thus these likely share a common origin. In contrast, *Pe19*.h2 contains the full intron and has a common origin with the avirulent haplotype 1 (Fig 8B). Consequently, the virulent genotype of *Pe19* has originated most likely from a mutation that already existed in the avirulent isolates and a novel mutation unique to *Pe19*.h2.

## Discussion

Filamentous plant pathogens evolve rapidly to overcome resistances of new crop varieties, often within a few growing seasons [5,6,9]. Resistance of crop varieties is often conferred by qualitative and monogenic effector triggered immunity [7]. Pathogens, in response, adapt their effector repertoire to avoid recognition and maintain virulence [1]. Host NLRs that recognize pathogen effectors have been extensively studied in a few model systems, on fungal pathogens mostly in the genus *Blumeria*, *Magnaporthe*, and *Leptosphaeria*, and in the oomycete pathogens in *Phytophthora* sp. [45]. However, little is known about the diversity of pathogen effector proteins and their evolution, especially in non-model plant pathogens [21,22]. To our knowledge, this study represents the most extensive genome assembly-based comparison of isolates within a single oomycete species. For the comparison, we constructed a pangenome graph with chromosome-level genome assemblies of 19 *P. effusa* isolates, each corresponding to a denominated race capable of overcoming spinach resistance genes. While the chromosome structure is highly conserved between these isolates, our analysis uncovered an open pangenome, indicating there is more variation to be discovered in the *P. effusa* population, mostly due to the extensive variation caused by TE activity. Variant calling revealed extensive differences in heterozygosity between isolates and between chromosomes within an isolate, which can be explained by the combination of sexual and asexual reproduction and by the loss of heterozygosity in specific genomic regions. Importantly, the fully phased effector repertoires allowed us to pinpoint candidate effectors that are variable between isolates and correlate with the breakage of specific spinach NLR resistance genes. These effector candidates are prime targets for downstream analysis to evaluate their contribution to virulence and function, as well as their potential mode of recognition.

In filamentous fungi, such as in *Verticillium dahliae*, *Fusarium oxysporum*, and *Magnaporthe oryzae*, large-scale chromosomal rearrangements and large accessory regions or chromosomes are often proposed to be the main drivers of genetic variation [46–53]. In contrast, the analysis of the 19 *P. effusa* isolate pangenome corroborates that the chromosome structure of *P. effusa* is highly conserved [32]. This conserved chromosome structure is similar to rust fungi, such as previously observed in the genus *Puccinia,* which are also diploids with various degrees of heterozygosity [54–56]. In the absence of large-scale structural variation and accessory chromosomes, smaller mutations (1 bp–10 kb), allelic variation, and recombination of isolates due to sexual reproduction are the main drivers of evolution [7,54,55].

Oomycetes, like rust fungi, have both sexual and asexual cycles [7,29,57]. Sexual recombination contributes to the evolution of these pathogens by chromosomal admixture between haplotypes, with some races in *Puccinia graminis* formed as a direct result of recombination [54]. Similarly, we provide evidence of recent recombination in five of the 19 *P. effusa* isolates, with seven more showing evidence of past recombination that had been followed by long periods of clonal reproduction. Clonal reproduction can lead to loss of alleles via loss of heterozygosity, which could allow the phenotypic expression of a recessive virulent allele due to the loss of a dominant avirulent allele, enabling isolates to overcome host resistance [7,54]. We observed loss of heterozygosity in multiple chromosomes of some *P. effusa* isolates, for example, in chromosome 9 in *Pe19* (Fig 4C). These patterns are consistent with genetic bottlenecks or selective sweeps, which can accelerate the fixation of advantageous alleles and reduce genetic diversity, often resulting in extended homozygous regions [58–60]. Such processes suggest that loss of heterozygosity events can accumulate and persist during extended clonal propagation, particularly under conditions that favor specific virulence traits, such as monocultures of resistant spinach varieties.

Oomycete RXLR effectors, most commonly in *Phytophthora* species, have been shown to suppress plant immunity, like AVR1 and PITG20303 of *P. infestans*, PpE18 of *P. parasitica*, PcAvr3a12 of *P. capsici*, and RxLR50253 of *Plasmopara viticola* [61–65]. RXLR effectors also trigger plant immunity, like AVR1 of *P. infestans* in R1 potato plants, multiple effectors with a WY domain in *Bremia lactucae* in lettuce, and Avr1b and AvrNb of *Phytophthora sojae* in *Nicotiana benthamiana* [20,66–70]. Here, we investigated effector variation in *P. effusa* isolates and its correlation with their virulence in different spinach varieties. In most of these cases, the candidate effector genes in the avirulent isolate are highly conserved, while the alleles in virulent isolates accumulate non-synonymous mutations that often result in frame shifts. The most prevalent mechanism that has been shown to avoid recognition are effector gene deletions or point mutations, as shown in *P. infestans* where the virulent allele of *PiAvr4* encodes a truncated protein caused by frameshift, or in *Plasmopara viticola* were multiple RXLR genes have been deleted as a result of structural variations [71,72]. Similarly, we here identify candidate effectors that can be associated with resistance breaking due to gene deletions and truncation of proteins. Nevertheless, we could not discover a case where a single event can be attributed to the virulent phenotype of all isolates, but resistance breaking could be linked to multiple independent evolutionary events. Additionally, we also observed isolates that have both alleles that we have linked to the avirulent effector genotype, thus the absence of the effector protein needs to be achieved via an alternative mechanism, possibly through repression of expression via gene silencing (e.g., *Pe4* and *Pe6* for *RPF3*) (Fig 5A). Epigenetic gene silencing to repress effector expression and avoid recognition in resistant crops has been previously demonstrated in *Avr1b* and *Avr3a* of *P. sojae* [73,74]. Thus, it is possible that both epigenetic and “conventional” mutational processes jointly contribute to the resistance breaking of spinach. However, the complex evolution and the extensive genomic variation between the isolates make it challenging to pinpoint the exact mutations that caused resistance breaking, which could be linked to other untested loci. To improve our ability to detect avirulence effectors and understand their evolution, the study of a much larger number of isolates with known (a)virulence phenotypes would be necessary. Alternatively, a small number of additional isolates could already provide better resolution for specific resistances by selecting multiple isolates with identical phenotypes or by re-sequencing isolates for which a new phenotype has emerged after their initial isolation. These results can then be used as the starting point for downstream functional analysis at the molecular level to test the avirulence of effectors in plant tissue, however the molecular tools are currently lacking [75].

Like many other downy mildews, the rapid evolution of *P. effusa* leads to the emergence of multiple resistance-breaking isolates, often in a single cultural season [24].Together with its obligate biotrophic nature, this poses a significant challenge to isolate, phenotype, maintain, and study this devastating pathogen [12,41,76]. The recent advancements in sequencing and in comparative genomics such as the here applied pangenome graphs now start to enable us to uncover the genomes of these pathogens, to differentiate between isolates and to link this variation to phenotypic differences, thereby providing effectors as prime candidates for future experimentations. The computational approach described here, will continue to uncover detailed mechanisms of the rapid evolution of these pathogens, perhaps leading to predictions of the upcoming emergence of new phenotypes.

## Methods

### *Peronospora effusa* denominated races

*P. effusa* races are assigned by selecting an isolate for its ability to create a unique infection pattern on a defined set of differential spinach lines (Fig 1A). The exact origin of the original isolates is unknown, but all of them have originated from the USA (S5 Table). The phenotyping and maintenance of *P. effusa* denominated races, and the development of differential spinach lines are handled by the International Seed Federation [77]. To retain their pathogenicity, stored material is refreshed every two years by reinfecting spinach lines with selective resistances, ensuring no cross contamination from other races [78]. After this multiplication, all isolates are then tested on the differential set of spinach line to ensure that their infection pattern remains stable. As it has been shown in other obligate biotrophic plant pathogens, what is described as an isolate could be a population of genetically distinct isolates with identical phenotypes, rather than a single genetically distinct isolate [79]. However, by creating chromosome-level assemblies and phasing with high-quality variants, a single most abundant genotype is analyzed for each isolate, which is also emphasized by the allele frequency distribution present for each isolate that demonstrates an expected allele frequencies for diploid isolates (S3 Fig). Moreover, based on our previous short-read data analysis we could not discover any multiallelic variation on the mitochondrial genomes of *P. effusa* isolates [29].

### *Peronospora effusa* infection on soil-grown spinach and spore isolation

Spinach plants were grown in potting soil (Primasta, the Netherlands) under long-day conditions (16-hour light, 21 °C). Two to three weeks post-germination, plants were inoculated with *P. effusa* by spraying them with a spore suspension in water using a spray gun. After inoculation, plants were kept under 9-hour light at 16 °C in humidified trays, where lids were sprayed with water and kept covered. Vents were opened after 24 hours, and lids were re-sprayed and resealed 7–10 days post-inoculation to maintain humidity and promote *P. effusa* sporulation.

For spore collection, sporulating leaves were placed in a glass bottle with tap water and shaken to release spores. The suspension was filtered through a 50-μm nylon mesh (Merck Millipore, USA) to remove large debris and then through an 11-μm nylon mesh using a vacuum pump to remove smaller contaminants. Spores retained on the filter were washed, scraped off, and stored at −80 °C for Oxford Nanopore sequencing.

### High-molecular-weight DNA extraction protocol

High-molecular-weight (HMW) DNA was isolated from *P. effusa* spores by grinding them into a fine powder in liquid nitrogen with 0.17–0.18 mm glass beads. The powdered spores were washed with cold sorbitol solution (100 mM Tris-HCl, 5 mM EDTA, 0.35 M sorbitol, 1% PVP-40, 1% β-mercaptoethanol, pH 8.0). Lysis was performed in extraction buffer (1.25 M NaCl, 200 mM Tris-HCl pH 8.5, 25 mM EDTA pH 8.0, 3% CTAB, 2% PVP-40, 1% β-mercaptoethanol) containing proteinase K and RNase A, incubated at 65 °C for 60 min with gentle inversion. Debris was pelleted by centrifugation. HMW DNA was purified using phenol/chloroform/isoamyl alcohol (IAA) and chloroform/IAA extractions, followed by an additional RNase treatment, purification with phenol/chloroform/IAA and chloroform/IAA, and isopropanol precipitation. DNA concentration and integrity were assessed using Nanodrop, Qubit, and Tapestation.

### Genome sequencing using Oxford Nanopore

We obtained long-read sequencing data for 13 *P. effusa* isolates with Oxford Nanopore sequencing technology (Oxford Nanopore, UK) at the USEQ sequencing facility (the Netherlands). We used a Nanopore PromethION flowcell (R9.4.1) for real-time sequencing and base-calling of the raw sequencing data was performed using Guppy (version 4.4.2; default settings).

### Genome assembly

To produce chromosome-level genome assemblies of 13 *P. effusa* isolates, we used the long-read Oxford Nanopore sequencing data. The reads were corrected, trimmed, and assembled using Canu (version 2.3) [80] with the following command:

canu -nanopore ${input_reads} genomeSize=58M corOutCoverage=40 mhapMemory=100g corMhapFilterThreshold=0.0000000002 mhapBlockSize=500 ovlMerThreshold=500
corMhapOptions=“--threshold 0.80 --num-hashes 512 --num-min-matches 3
--ordered-sketch-size 1000 --ordered-kmer-size 14 --min-olap-length 800 --repeat-idf-scale 50“

input_reads: nanopore long-read in fasta or fastq format

### Scaffolding assemblies and closing gaps

The assemblies were scaffolded to full chromosomes with ragtag scaffold (v. 2.1.0, default settings) [81] using as reference the most closely related genome assembly from the previous assembled *P. effusa* isolates (*Pe1*, *Pe4*, *Pe5*, *Pe11*, *Pe14*, and *Pe16*) [32]. Gaps in the scaffolded chromosomes were closed with FinisherSC (version 2.1; default settings) [82], and the scaffolded assemblies were corrected for SNPs using Illumina short-reads with four rounds of Pilon (version 1.23; --diploid, --fixbases) [83]. The used Illumina short-reads were sequenced in our previously published work [32].

### Transposable element and genome annotation

The combined *P. effusa* TE library created from *Pe1*, *Pe4*, *Pe5*, *Pe11*, *Pe14*, and *Pe16* [32] was used to annotate and soft-mask the genomes using RepeatMasker (version 4.1.2 -e rmblast -xsmall -s -nolow) [84]. We utilized the RNAseq short-read data from isolates *Pe1*, *Pe5*, *Pe11*, and *Pe16* that were isolated from *P. effusa* spores and infected spinach leaves and were sequenced in our previously published work [32]. The soft-masked genomes and RNAseq data were used for structural gene prediction and functional annotation with the funannotate pipeline (version 1.8.7) (--stranded no --jaccard_clip, --max_intronlen 600, --alt_transcripts 0.3) [85] as described previously [32].

### Secretome and effector prediction

To detect secreted proteins and effector candidates, we applied a previously published approach [32]. In short, we used the genes annotated by funannotate and additional open reading frames, encoding at least 70 amino acids, to predict the secretome using the Predector pipeline (v. 1.2.6, default settings) [86]. The secreted proteins were then screened to detect the presence of the conserved motifs described in RXLR and Crinkler oomycete effectors in the canonical and divergent forms. The search was performed using regular expression with the EffectR package for R [87] and sequence profile searches using HMMER v3.3 [88].

### Pangenome graphs and common annotation

A pangenome graph was built per chromosome and then merged in a final pangenome graph following the Minigraph-Cactus Pangenome Pipeline, HPRC Graph (step-by-step): Splitting by Chromosome (version 2.6.4, --filter 0 --vcf full --gfa full) [37]. This results in one, unfiltered pangenome graph of all 17 core chromosomes of all 19 isolates that was used in the downstream analysis. Pangenome graphs from Minigraph were visualized with bandage (version 0.8.1) [40].

The hal output of the pangenome graph, the gene annotation, the annotated protein sequences, and the RNAseq coverage for each *P. effusa* isolate were used as input to collectively reannotate genes with the Comparative-Annotation-Toolkit (version 2.2.1, --augustus --augustus-cgp --assembly-hub --filter-overlapping-genes) [89]. To filter ORFs of unknown origin, we removed from the annotation genes that were not characterized as protein-coding (gene_biotype=protein_coding) or had no “alternative_source_transcripts” in the gff line of the tRNA.

### Synteny-based gene orthogroups

Synteny-based gene orthogroups were created based on our previously published approach and the code is available on GitHub [32]. In short, the gfa file of pangenome graph was parsed and annotated with the genes of each isolate. The genes were then assigned to orthogroups based on their syntenic localization along the graph. In total, this resulted in 15,739 orthogroups with a single gene for each isolate represented in each orthogroup. Each orthogroup is characterized as core (19 isolates), accessory (2–18 isolates), or unique (1 isolate) based on the number of isolates represented in each orthogroup. These assigned orthogroups determine the gene presence absence for all isolates.

### Saturation plots

Saturation plots are created based on our previously published approach [32]. Briefly, the variation between the isolates was visualized by making all the possible comparison for combinations of one to all 19 isolates. We characterized each count based on the number of isolates represented, as core (all isolates in the comparison), unique (only one isolate for comparisons of two isolates or more), or accessory. The line is drawn on the mean for each combination (one to 19) and the range of all calculations are shown by the shadow behind each line.

### Haplotype assignment

Each variable node, i.e., each node that is not present in at least one isolate, in the parsed pangenome file was assigned a haplotype. Nodes were assigned as a unique if they only occurred in one isolate. The rest of the nodes were recursively assigned to a haplotype, based on the isolates represented in the node. For a given window size, we calculated the haplotype that is most abundant by length. If the total length of that haplotype is above 5% of the windows size, the whole window is labeled to belong to that haplotype. Otherwise, the window is characterized as core. The alignment of the region is visualized in python using pandas and matplotlib [90].

### Variant calling with nanopore reads

Nanopore read of each isolate were mapped to the respective genome assemblies using minimap2 (v. 2.21, -ax map-ont) [91]. Variant calling of short variants and the phasing of the nanopore reads was performed with the PEPPER-Margin-DeepVariant pipeline, following Oxford Nanopore R9.4.1 variant calling workflow [Using singularity] (v. 0.8) [42]. The following command was used:

apptainer exec pepper_deepvariant_r0.8.sif run_pepper_margin_deepvariant call_variant

-b “${nanopore_bam}” -f “${ref_fasta}” -p “PePEPPER_Margin_DeepVariant”
-o “${output_dir}” --ont_r9_guppy5_sup --phased_output
--pepper_min_mapq 10 --dv_min_mapping_quality 10
--margin_haplotag_model allParams.haplotag.ont-r94g422.json
--margin_phase_model allParams.phase_vcf.ont.json

nanopore_bam: nanopore reads mapped to the reference assembly

ref_fasta: reference assembly in fasta format

output_dir: location where the output will be written

parameter files were downloaded from the GitHub page of the tool margin: margin/params/phase at master · UCSC-nanopore-cgl/margin

The bam file of phased nanopore reads was then used to perform variant calling for phased long variants using Sniffles2 (v. 2.3.3, --phase). The phased insertions and deletions discovered by Sniffles2 were merged with the phased short variants from PEPPER by giving priority to a deletion when it overlapped with other SNPs in the same phase.

### Phasing of genomes and genes

The merged vcf file of phased variation was used to create two copies of each genome assembly, one for each phase with bcftools consensus (v. 1.16, -e ‘ALT~“<.*>”’) [92]. Individual chromosome of the phased copies from the genome assemblies were used to create phased pangenome graphs as described above, by adding the haplotype after the isolate name (e.g., *Pe5*.1 and *Pe5*.2) [37].

### Effector association with virulence

For each effector orthogroup, we calculated the protein sequence similarity of all proteins compared to the longest allele. We searched for potential associations between protein variation between effector in an orthogroup and avirulence. To narrow down our search we filtered for orthogroups that combine the following characteristics: (i) all avirulent isolates have at least one allele with 85% protein sequence similarity; (ii) the group of virulent isolates have at least one isolate with both alleles with less than 85% sequence similarity. Heatmaps of the outcome were split into virulent and avirulent groups and visualized in python using matplotlib and seaborn (S9–S11 Figs) [90,93]. From these tables, we prioritized four cases for their best association with the virulent and avirulent groups (S6 Table).

### Splits tree

We performed variant calling, using the genome assembly of *Pe1* as a reference, and the short-read data of 19 *P. effusa* isolates. The short reads were aligned using bwa-mem2 (version 2.2.1, default settings) [94] and a joint VCF file was generated with both variant and invariant sites with GATK (version 4.4.0.0, GenotypeGVCFs -all-sites) [95]. The single-nucleotide variants were transformed into a distance matrix with PGDSpider (version 2.1.1.5) [96], which was then used to construct a decomposition network using the Neighbor-Net algorithm with SplitsTree (version 4.17.0) [97]. We calculated the branch confidence of the network using 1,000 bootstrap replicates.

### Visualizations

Whole-genome alignments of the 19 genomes were performed and visualized using GENESPACE (minPepLen 50, blkSize 10, minGenes2plot 10) [98]. All other visualizations were created in python using matplotlib and seaborn [93].

## Supporting information

S1 Glossary Glossary of terms. (DOCX)

S1 Fig Whole-genome alignments of genomes based on sequence similarity and on relative position of protein-coding genes. Comparison of our 19 chromosome-level genome assemblies for *Peronospora effusa* revealing highly conserved chromosome structure (Data in Zenodo).
(TIF)

S2 Fig The structure and variation of the pangenome graph of 19 *Peronospora effusa* isolates. **A.** Histogram of the node degree, i.e., the number of connections, of each node of the pangenome graph (Data in S7 Table). **B.** Histogram of the sequence length of each node of the pangenome graph up to a max length of 40 kb. **C.** Bar plot of the percentage of the genome size that is core, accessory, or unique for each isolate and the pangenome (Data in S7 Table).
(TIF)

S3 Fig Allele frequency has a normal distribution around the 0.5 frequency, as expected for a diploid organism. Histogram of allele frequency of the combined heterozygous variants from PEPPER and Sniffles2. **A.** The distribution closest to normal for *Pe7* (Data in S7 Table). **B.** The distribution that deviates most from normal for *Pe16* (Data in S7 Table).
(TIF)

S4 Fig Phylogeny of *Peronospora effusa* haplotypes bases on chromosome 9. Dendrogram based on the hierarchical clustering of the accessory nodes of the phased chromosome 9 from 19 *P. effusa* isolates (Data in S7 Table).
(TIF)

S5 Fig The similarity of the 17 chromosomes of 19 *Peronospora effusa* isolates. The haploid chromosomes are coloured based on the best match to a different isolate. The chromosomes where split in an 800 kb window to offer an overview of all the chromosomes in a single figure. As targets we selected the 10 isolates with most contribution to the genomic diversity based on the method described in Fig 4A (Data in Zenodo).
(TIF)

S6 Fig Histograms of the protein sequence identity of haplotypes after phasing. **A.** Histogram of *Pe3* with the least number of phased variants applied to genes (Data in S7 Table). **B.** Histogram of *Pe11* with the most phased variants applied to genes (Data in S7 Table).
(TIF)

S7 Fig Enrichment of subgroups of protein for variation between alleles. *P*-values were calculated using Fisher’s exact test with Benjamini-Hochberg correction. **A.** Heatmap of enrichment for any sequence variation between protein alleles. **B.** Heatmap of enrichment for protein deletion between protein alleles.
(TIF)

S8 Fig Comparison of the protein sequence similarity between 19 *Peronospora effusa* isolates for different groups of genes. Line plots for the cumulative number of genes that have orthologs in all 19 isolates with a minimum protein identity. Plots are made for all genes, effectors, and effector outside physical clusters (Data in S7 Table).
(TIF)

S9 Fig Heatmap of the protein sequence similarity to the longest effector of each orthogroup, which includes both alleles of all isolates. The heatmap is separated by the (a)virulent phenotype against the spinach with the resistance gene *RPF3*. The orthogroup that we selected to prioritize is highlighted (Data in Zenodo).
(TIF)

S10 Fig Heatmap of the protein sequence similarity to the longest effector of each orthogroup, which includes both alleles of all isolates. The heatmap is separated by the (a)virulent phenotype against the spinach with the resistance gene *RPF4*. The orthogroups that we selected to prioritize are highlighted (Data in Zenodo).
(TIF)

S11 Fig Heatmap of the protein sequence similarity to the longest effector of each orthogroup, which includes both alleles of all isolates. The heatmap is separated by the (a)virulent phenotype against the spinach with the resistance gene *RPF11*. The orthogroup that we selected to prioritize is highlighted (Data in Zenodo).
(TIF)

S1 Table Metrics of Nanopore long-read sequencing for 19 *Peronospora effusa* isolates. (XLSX)

S2 Table Size of the assembled chromosomes for 19 *Peronospora effusa* isolates. (XLSX)

S3 Table Metrics of the structural annotation (a; genes, b; effectors, c; repeats) of the assemblies of 19 *Peronospora effusa* isolates. (XLSX)

S4 Table Number of phased variants overlapping with genic regions and number of genes that were phased for 19 *Peronospora effusa* isolates. (XLSX)

S5 Table Details about the origin of the here sequenced *Peronospora effusa* isolates. (XLSX)

S6 Table Details of the association of resistance genes with effector orthogroups. (XLSX)

S7 Table Data underlying multiple figures. (XLSX)

## Abbreviations

BUSCO: Benchmarking Universal Single-Copy Ortholog
HMW: high-molecular-weight
IAA: isoamyl alcohol
NLRs: nucleotide-binding leucine-rich repeat receptors
SNPs: single-nucleotide polymorphisms
TE: transposable element
