# Supplementary material for: Pangenome graph analysis reveals evolution of resistance breaking in spinach downy mildew
Source: PLoS Biol. 2026 Jan 20;24(1):e3003596. doi: 10.1371/journal.pbio.3003596 (PMC12844528; doi:10.1371/journal.pbio.3003596)
Supplement: S1 Glossary — (DOCX) [file pbio.3003596.s001.docx]

## Glossary of terms

**Accessory region**A genomic segment present in some isolates but absent in others. Accessory regions often contain genes linked to adaptation, such as effectors.

**Core region**
Genomic sequences shared by all isolates in the study. Represents the conserved part of the genome.

**Unique region**
A genomic segment found only in a single isolate. Often reflects recent mutations or structural changes.

**Haplotype**
One of the two sets of chromosomes in a diploid organism. Each haplotype carries a unique combination of alleles.

**Haplotype block**
A contiguous region of DNA where alleles are inherited together. Used to study recombination and genetic variation.

**Loss of heterozygosity**
A genetic event where one allele at a locus is lost, making the region homozygous.

**Pangenome (sequence resolved)**
The complete genome content across all isolates of a species, including core, accessory, and unique regions.

**Pangenome graph**
A graph-based representation of the pangenome, where nodes represent sequences and edges represent connections between them. Used to visualize variation across isolates.

**Phased genome / phasing**
The process of separating the two haplotypes in a diploid genome to study allele-specific variation.

**Selective sweep**
A process where a beneficial mutation becomes fixed in a population, reducing genetic diversity in surrounding regions.

**Transposable element (TE)**
A mobile DNA sequence that can change its position within the genome, often contributing to genome expansion and variation.

**Orthogroup**

A set of genes across isolates that are considered orthologous (derived from a common ancestor).

**Synteny**

Conservation of gene order across genomes.

**Variant calling**

Computational process to identify genetic differences (SNPs, insertions, deletions) between sequences.
