# Supplementary figures and images for: Pangenome graph analysis reveals evolution of resistance breaking in spinach downy mildew
Source: PLoS Biol. 2026 Jan 20;24(1):e3003596. doi: 10.1371/journal.pbio.3003596 (PMC12844528; doi:10.1371/journal.pbio.3003596)

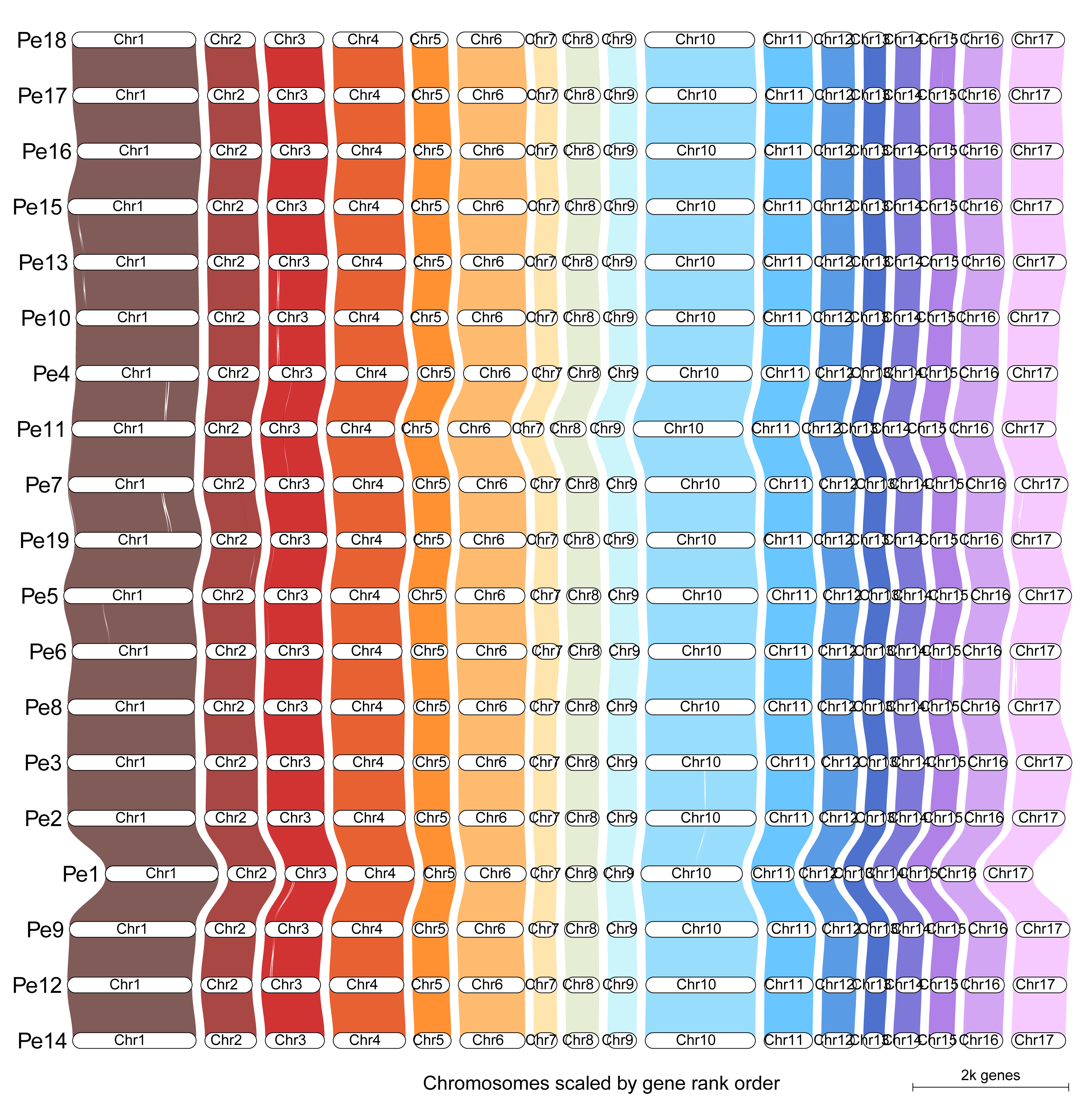

Supplement: S1 Fig — Comparison of our 19 chromosome-level genome assemblies for Peronospora effusa revealing highly conserved chromosome structure (Data in Zenodo). (TIF) [file pbio.3003596.s002.tif]

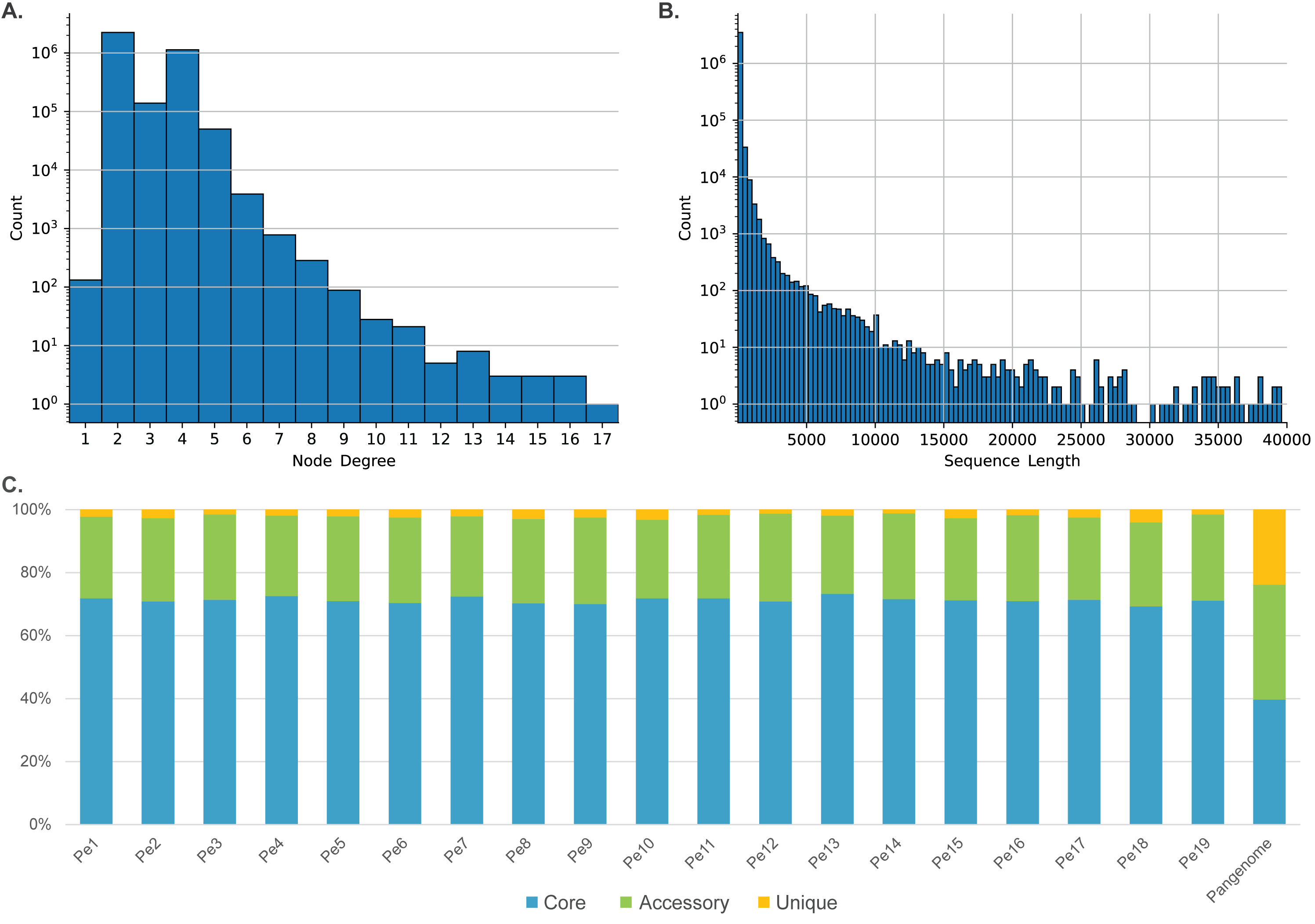

Supplement: S2 Fig — A. Histogram of the node degree, i.e., the number of connections, of each node of the pangenome graph (Data in S7 Table). B. Histogram of the sequence length of each node of the pangenome graph up to a max length of 40 kb. C. Bar plot of the percentage of the genome size that is core, accessory, or unique for each isolate and the pangenome (Data in S7 Table). (TIF) [file pbio.3003596.s003.tif]

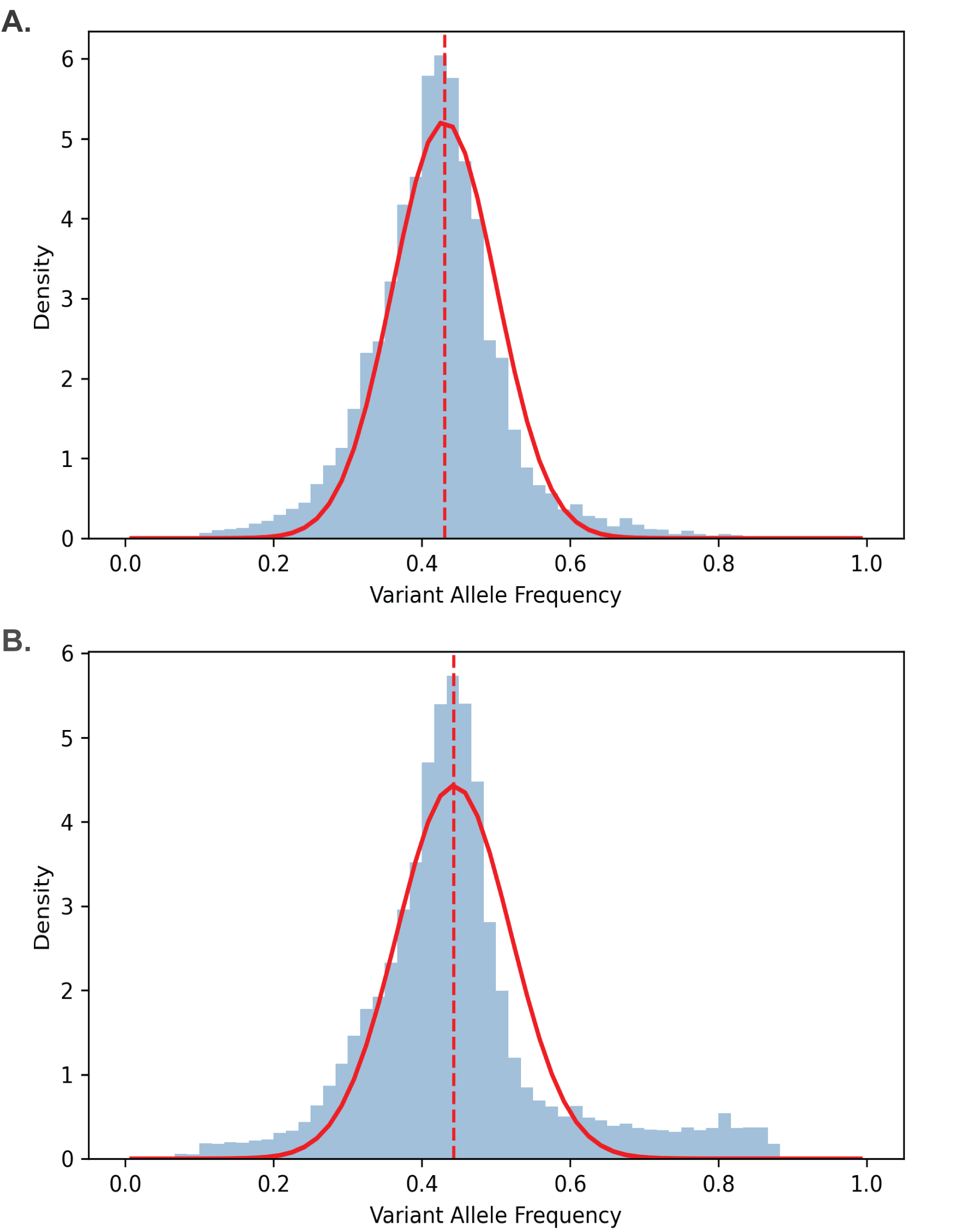

Supplement: S3 Fig — Histogram of allele frequency of the combined heterozygous variants from PEPPER and Sniffles2. A. The distribution closest to normal for Pe7 (Data in S7 Table). B. The distribution that deviates most from normal for Pe16 (Data in S7 Table). (TIF) [file pbio.3003596.s004.tif]

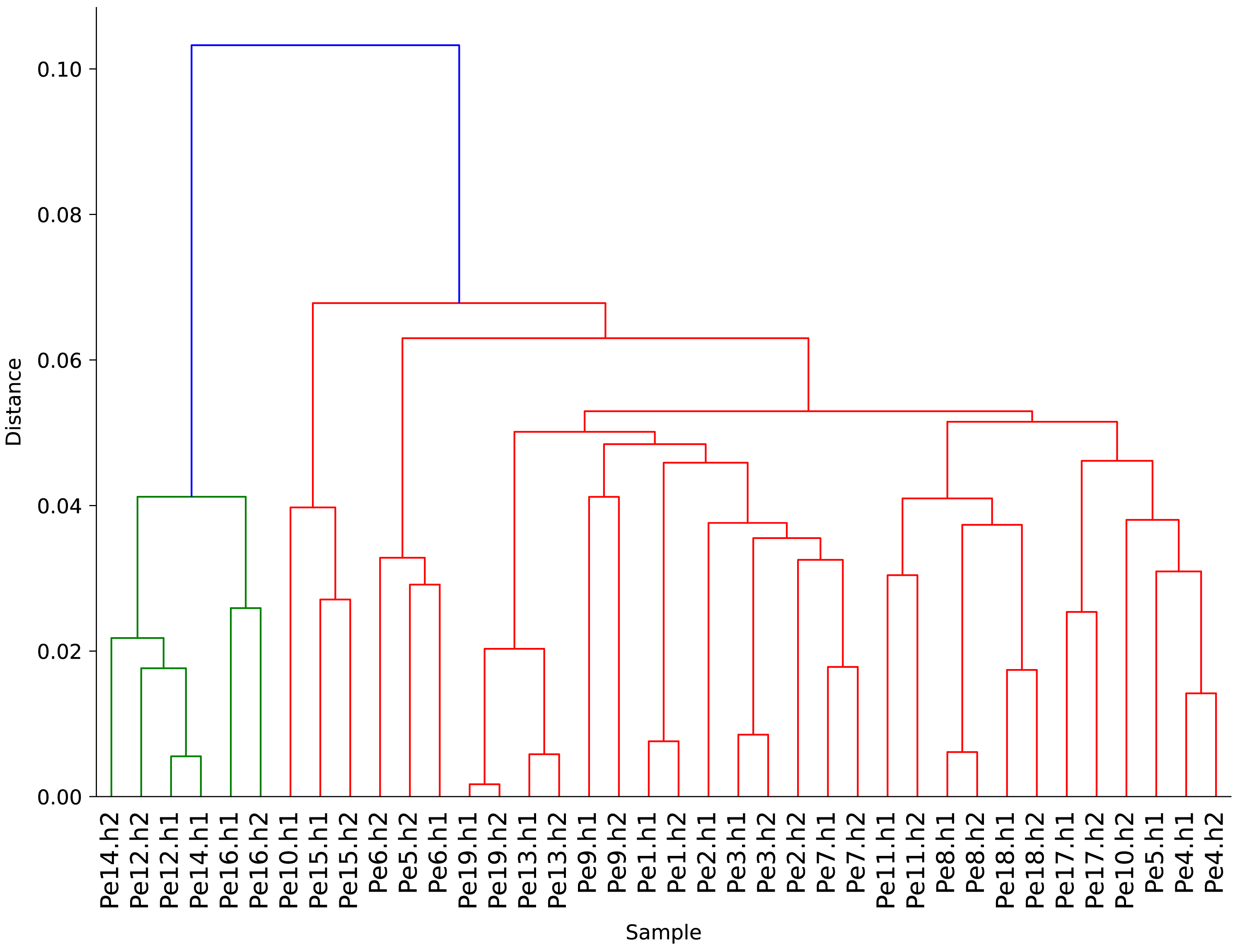

Supplement: S4 Fig — Dendrogram based on the hierarchical clustering of the accessory nodes of the phased chromosome 9 from 19 P. effusa isolates (Data in S7 Table). (TIF) [file pbio.3003596.s005.tif]

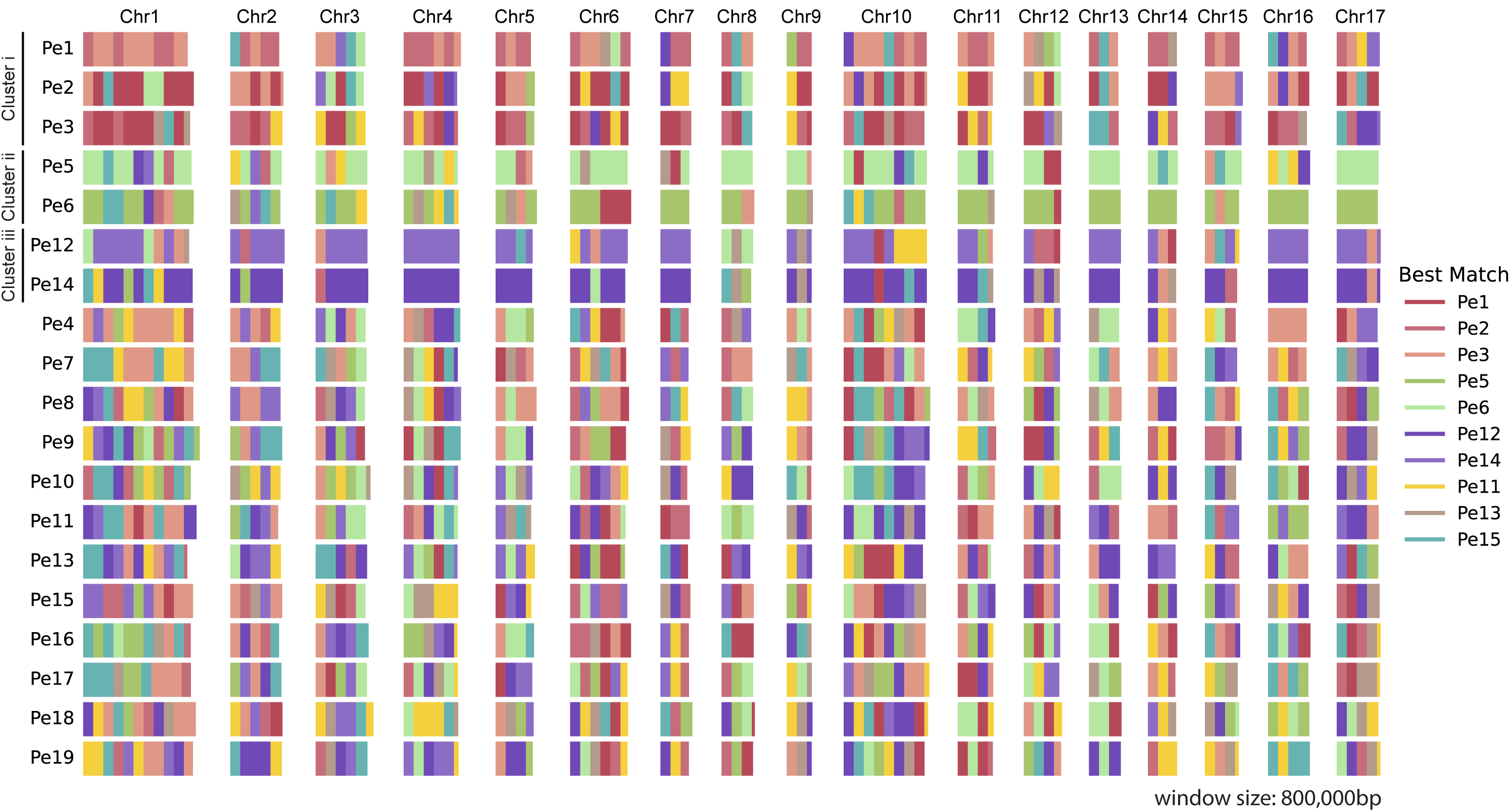

Supplement: S5 Fig — The haploid chromosomes are coloured based on the best match to a different isolate. The chromosomes where split in an 800 kb window to offer an overview of all the chromosomes in a single figure. As targets we selected the 10 isolates with most contribution to the genomic diversity based on the method described in Fig 4A (Data in Zenodo). (TIF) [file pbio.3003596.s006.tif]

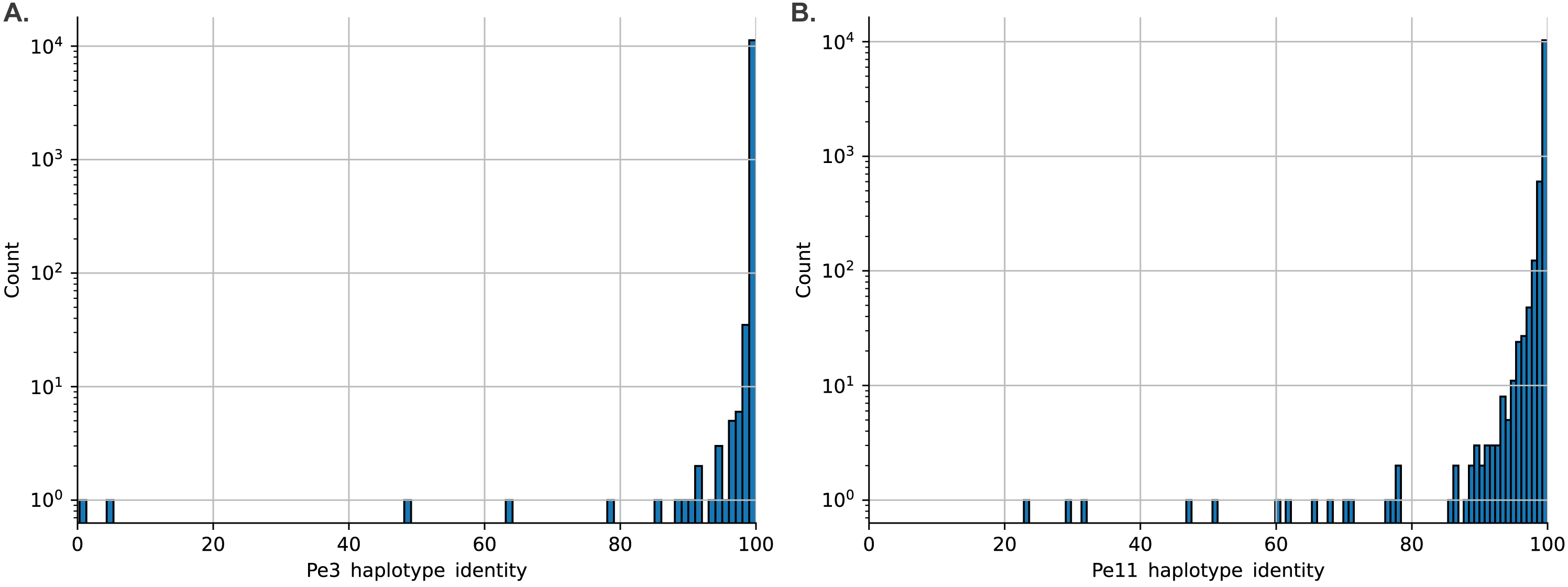

Supplement: S6 Fig — A. Histogram of Pe3 with the least number of phased variants applied to genes (Data in S7 Table). B. Histogram of Pe11 with the most phased variants applied to genes (Data in S7 Table). (TIF) [file pbio.3003596.s007.tif]

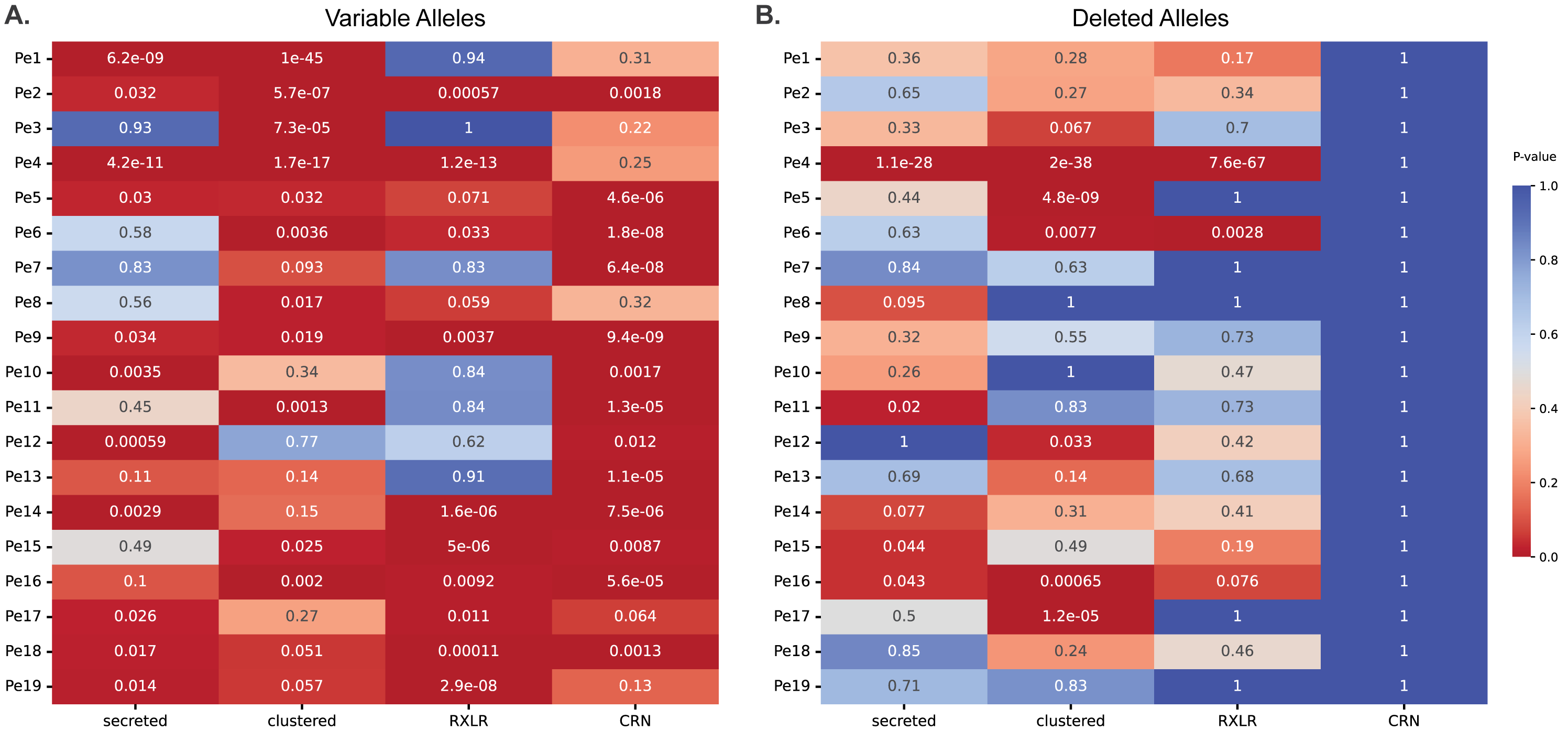

Supplement: S7 Fig — P-values were calculated using Fisher’s exact test with Benjamini-Hochberg correction. A. Heatmap of enrichment for any sequence variation between protein alleles. B. Heatmap of enrichment for protein deletion between protein alleles. (TIF) [file pbio.3003596.s008.tif]

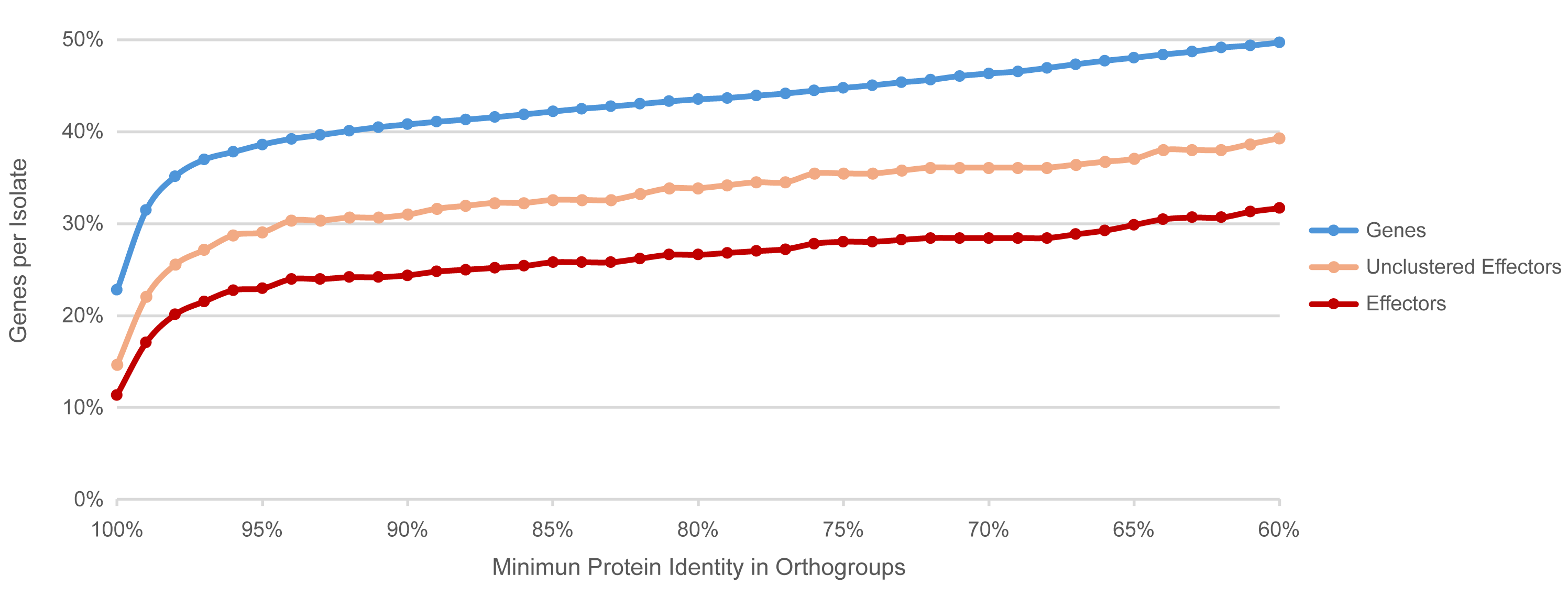

Supplement: S8 Fig — Line plots for the cumulative number of genes that have orthologs in all 19 isolates with a minimum protein identity. Plots are made for all genes, effectors, and effector outside physical clusters (Data in S7 Table). (TIF) [file pbio.3003596.s009.tif]

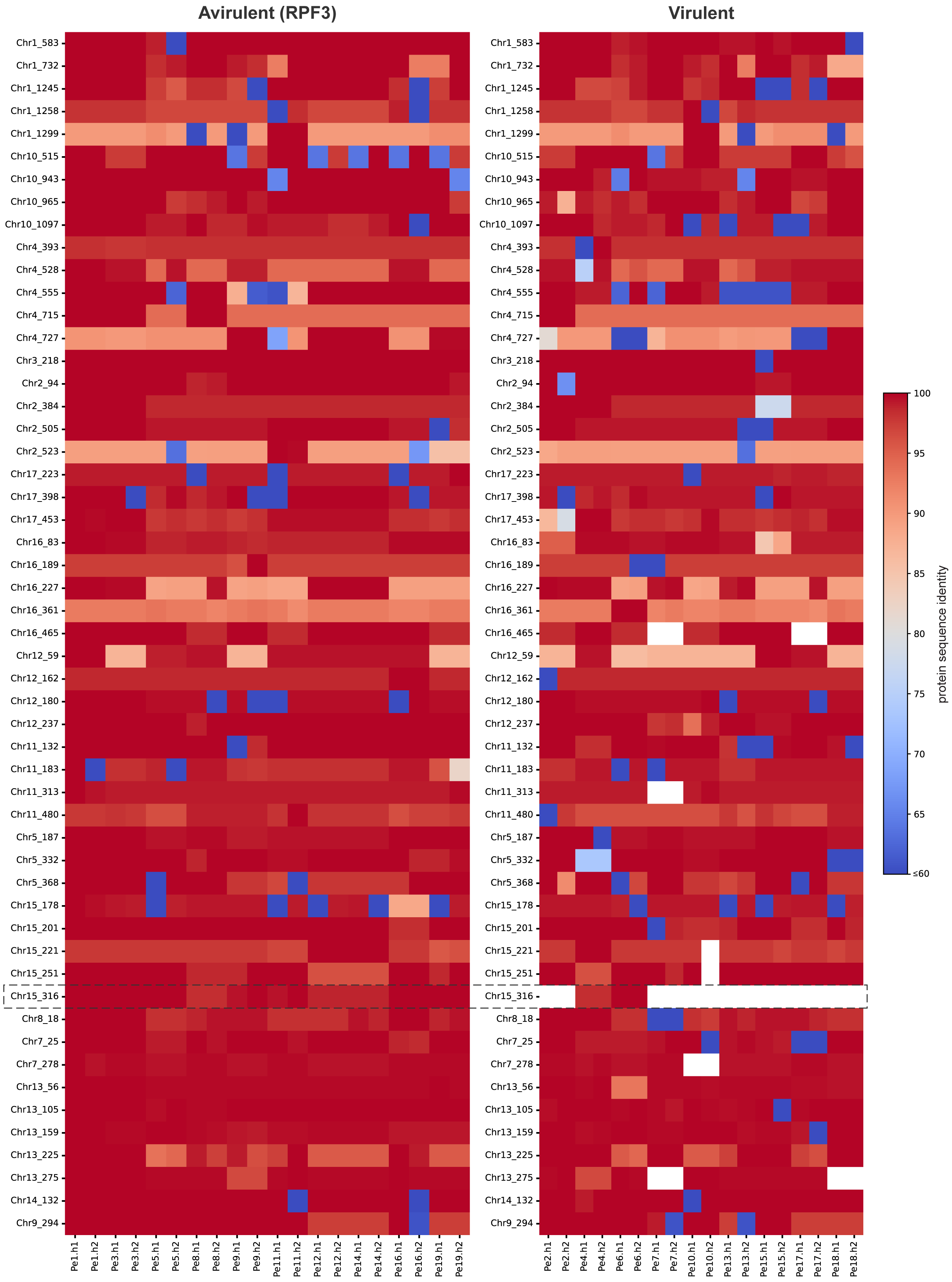

Supplement: S9 Fig — The heatmap is separated by the (a)virulent phenotype against the spinach with the resistance gene RPF3. The orthogroup that we selected to prioritize is highlighted (Data in Zenodo). (TIF) [file pbio.3003596.s010.tif]

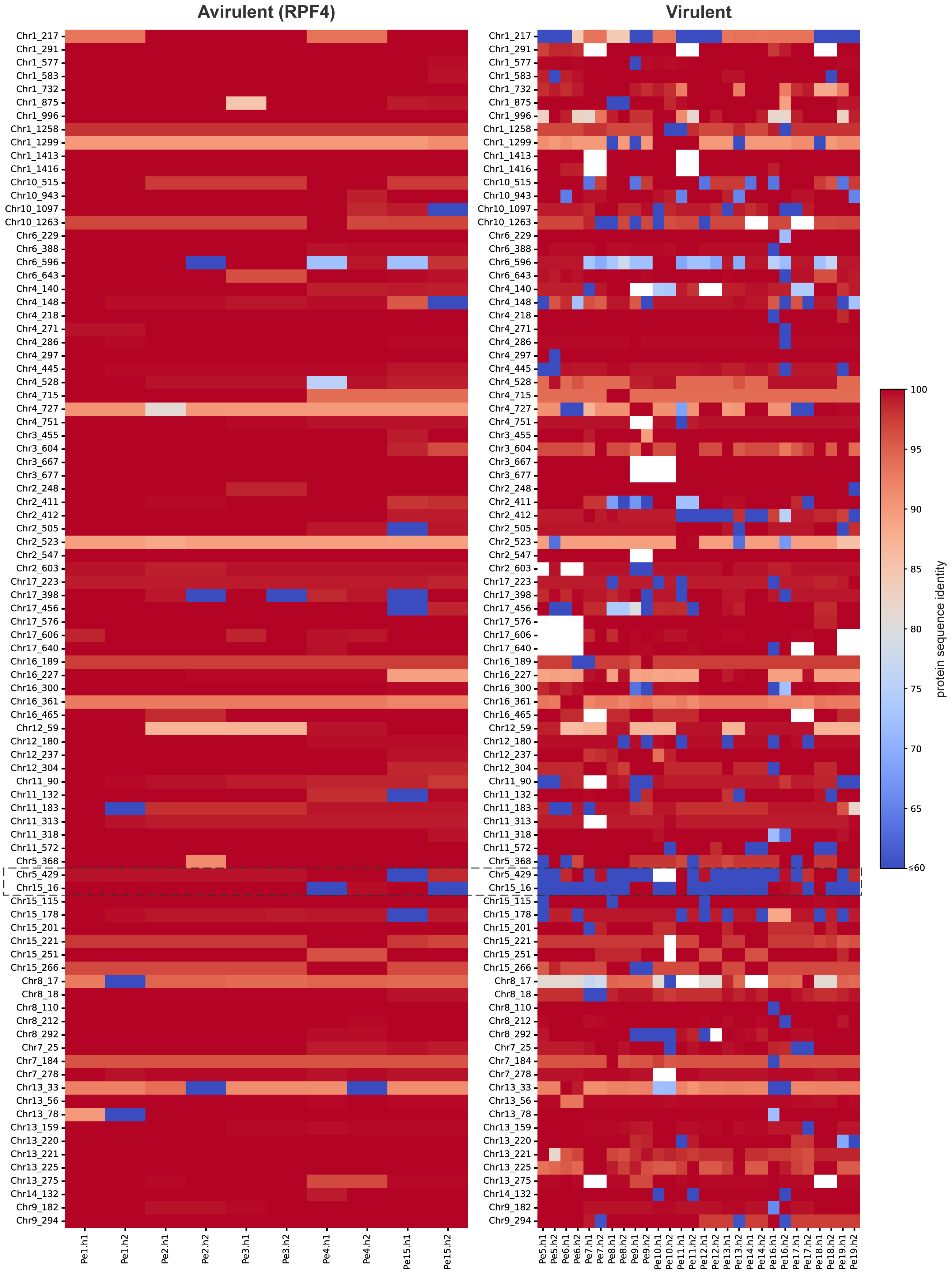

Supplement: S10 Fig — The heatmap is separated by the (a)virulent phenotype against the spinach with the resistance gene RPF4. The orthogroups that we selected to prioritize are highlighted (Data in Zenodo). (TIF) [file pbio.3003596.s011.tif]

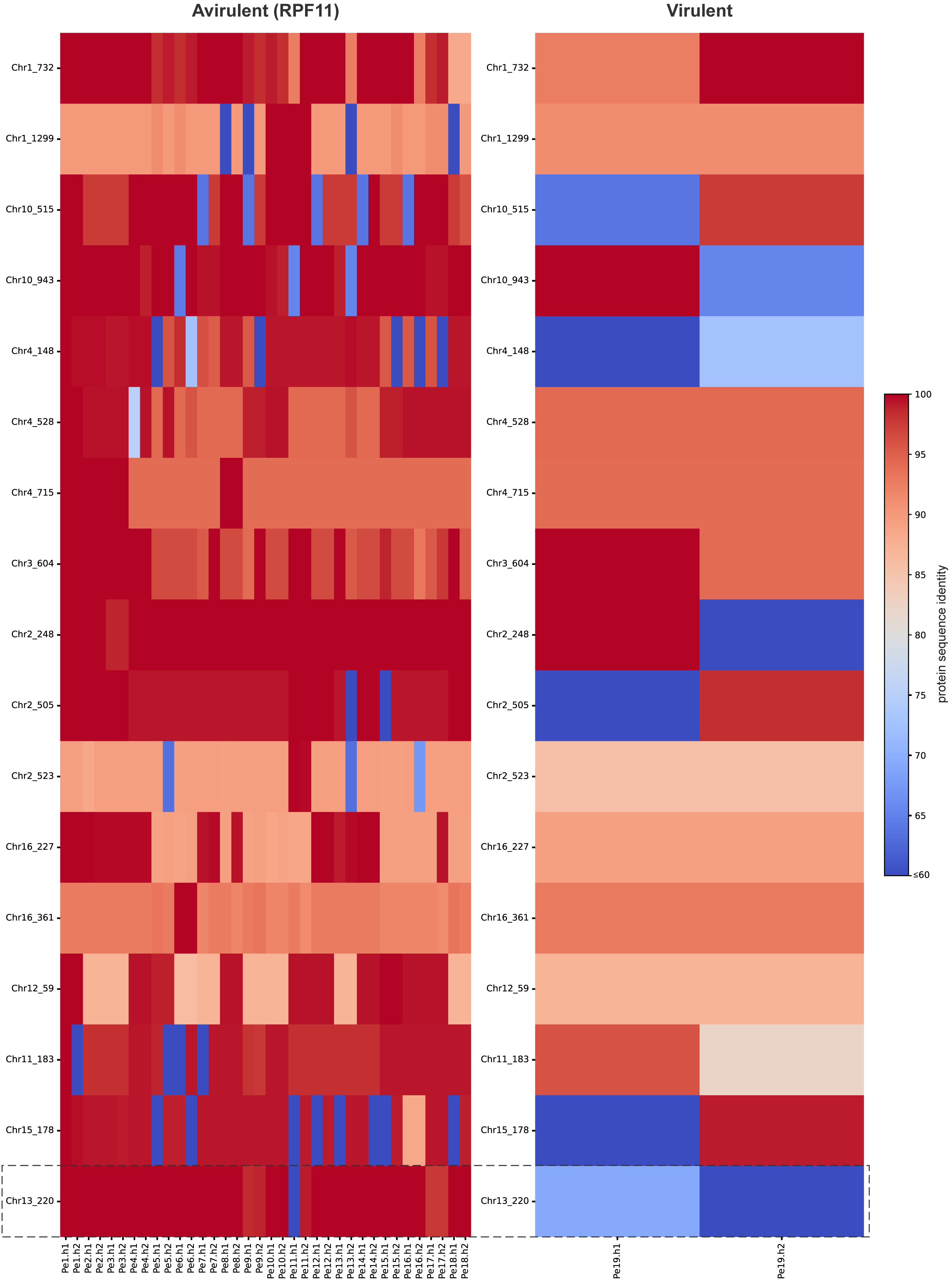

Supplement: S11 Fig — The heatmap is separated by the (a)virulent phenotype against the spinach with the resistance gene RPF11. The orthogroup that we selected to prioritize is highlighted (Data in Zenodo). (TIF) [file pbio.3003596.s012.tif]
